# New acorane-sesequiterpenes and anti-retinoblastoma constituents from the marine algicolous fungus *Trichoderma harzianum* NTU2180 guided by molecular networking strategy

**DOI:** 10.1186/s40529-024-00449-5

**Published:** 2025-01-14

**Authors:** Andrea Gu, Fan-Li Lin, Chung-Kuang Lu, Tz-Wei Yeh, Yih-Fung Chen, Ho-Cheng Wu, Tzong-Huei Lee

**Affiliations:** 1 Institute of Fisheries Science, College of Life Science, National Taiwan University, No. 1, Sec. 4, Roosevelt Rd., Da’an Dist, Taipei, 106319 Taiwan (R.O.C.); 2 School of Pharmacy, College of Pharmacy, Kaohsiung Medical University, No. 100, Shiquan 1st Rd., Sanmin Dist., Kaohsiung, 807378 Taiwan; 3 Department of Pharmacology, School of Post-Baccalaureate Medicine, College of Medicine, Kaohsiung Medical University, Kaohsiung, 807378 Taiwan; 4 Department of Medical Research, Kaohsiung Medical University Hospital, Kaohsiung, 807378 Taiwan; 5 National Research Institute of Chinese Medicine, Ministry of Health and Welfare, Taipei, 112304 Taiwan; 6 Graduate Institute of Natural Products, College of Pharmacy, Kaohsiung Medical University, Kaohsiung, 807378 Taiwan; 7 Department of Medical Research, Kaohsiung Medical University Hospital, Kaohsiung, 80756 Taiwan (R.O.C.)

**Keywords:** *Ex vivo* anti-angiogenic activity, Feature-based molecular networking (FBMN), OSMAC approach, Retinoblastoma, *Trichoderma harzianum* NTU2180, Triterpenoid

## Abstract

**Background:**

*Trichoderma* species, known as biocontrol agents against plant diseases, contain diverse compounds, especially terpenoids, with various bioactivities. To facilitate the exploration of bioactive secondary metabolites of *Trichoderma harzianum* NTU2180, the OSMAC approach MS/MS molecular networking was applied in the current study.

**Results:**

The feature-based molecular networking (FBMN) analysis showed that *T. harzianum* NTU2180 fermented on germinated brown rice (GBR) produced more terpenoids. Here, two new acorane-sesequiterpenes, trichospirols A (**1**) and B (**2**), and 12 known compounds (**3** − **14**) were isolated from the EtOAc layer of *T. harzianum* NTU2180 fermentation on GBR. Structures of these compounds were determined through NMR, UV, IR, and MS analyses. The absolute configuration of trichospirols A (**1**) was also elucidated by x-ray with Cu K-α radiation. Among them, six compounds (**1**, **2**, **3**, **4**, **5**, and **11**) were annotated as terpenoids by the NPClassifier on FBMN. 5-Hydroxy-3-hydroxmethyl-2-methyl-7-methoxychromone (**7**) and ergosterol peroxide (**11**) showed significant anti-angiogenic activity in ex vivo experiments with respective 0.57 ± 0.12- and 0.20 ± 0.12-fold changes. In addition, compound **11** displayed cytotoxicity against Y79 retinoblastoma cells with IC_50_ value of 35.3 ± 6.9 µM.

**Conclusions:**

The current study utilizes FBMN concept with OSMAC approach to accelerate the exploration of potential metabolites of the fungus *Trichoderma harzianum* NTU2180. Through a series of FBMN-guided isolation and purification, two new acorane-sesequiterpenes and 12 known compounds were obtained. The ex vivo and in vitro experiments were evaluated to assess anticancer isolates. It is worth noting that compound **11** was identified as a dual inhibitor targeting both angiogenesis and proliferation of retinoblastomas. Altogether, the results revealed the novel potential of *T. harzianum* for developing natural therapeutics against retinoblastomas.

**Supplementary Information:**

The online version contains supplementary material available at 10.1186/s40529-024-00449-5.

## Background

Natural products (NPs) have long been recognized as an important resource for drug discovery because of their diversity and complexity (Atanasov et al. 2021). Marine fungi have garnered increasing attention because they possess various pharmacological potentials. *Trichoderma* species, renowned fungi belonging to the Moniliaceae family (Ascomycetes, Hypocreales), began to draw scientific attention and gradually captivated the public’s interest. To the present, *Trichoderma* sp. have found extensive applications in agriculture, serving as biocontrol agents against diverse plant diseases. They are also utilized as sustainable bio-organisms to promote plant growth, contribute to natural decomposition, and engage in bioremediation efforts (Bai et al. 2023). Previous investigations of *Trichoderma* sp. demonstrated that secondary metabolites from *Trichoderma* possess diverse and promising bioactivities, including neuroprotective (Fang et al. 2017), anti-inflammatory (Li et al. 2021), antimicrobial (Ghisalberti et al. 1993; Leelavathi et al. 2014), cytotoxic (Chen et al. 2015), anticancer (Salim et al. 2020; Saravanakumar et al. 2021), antioxidant (Saravanakumar et al. 2021), and antiviral (Qian-Cutrone et al. 1996) activities. Many types of secondary metabolites have been isolated from *Trichoderma*, such as peptaibiotics, siderophores, diketopiperazines-like gliotoxin and gliovirin, polyketides, terpenes, pyrones, and isocyane metabolites (Zeilinger et al. 2016). Among these, terpenoids, especially sesquiterpenes, are the predominant secondary metabolites of *Trichoderma* (Bai et al. 2023). Several types of *Trichoderma* sesquiterpenes have been reported, including acorane, bisabolane, botryane, cadinane, carotane, cyclonerane, driman, and trichothecene types (Bai et al. 2023). These *Trichoderma* terpenoids exhibit diverse pharmacological effects due to their antibacterial, antifungal, and cytotoxic activities (Bai et al. 2023). Therefore, screening terpenoid-enriched fermentation processes is required to discover bioactive lead compounds.

Traditionally, the conventional approach for fungal metabolites analysis was bioassay-guided isolation and identification. However, employing this method may result in the re-identification of known compounds that lack a comprehensive overview of the metabolite profile. Hence, there is a need for a systematic analytical strategy to streamline the fungal metabolite analytical process, enabling the discovery of potential entities. Ultra high-performance liquid chromatography–tandem mass spectrometry (UHPLC-MS/MS) is a modern metabolomics research method for finding nonvolatile metabolites (Perez de Souza et al. 2021). However, metabolite identification and structure elucidation persist as challenges in untargeted MS/MS-based metabolomic profiling. In recent years, computational interpretation of MS/MS data has been introduced into metabolomics analyses to obtain a comprehensive view of complex mixtures. Molecular networking (MN) is a computational approach for grouping metabolites based on MS/MS spectral similarities (Quinn et al. 2017). The web-based platform Global Natural Products Social Molecular Networking (GNPS) can develop visualized structural relationships among molecules (Wang et al. 2016). Molecules with similar MS/MS spectra and analogous fragments will construct interconnected molecular networks. Furthermore, an advanced MN tool, feature-based MN (FBMN), is used to analyze fragment spectra to distinguish isomers, facilitate spectrum annotation, and include relative quantitative data (Nothias et al. 2020). Additionally, use of the software SIRIUS (Duhrkop et al. 2019) with CSI: FingerID (Duhrkop et al. 2015) and CANOPUS (Duhrkop et al. 2021), combined with FBMN file output from the GNPS environment in Cytoscape (Shannon et al. 2003) software, enables visualization of advanced annotation and compound class predictions. Therefore, by integrating different cultural methods of fungal fermentation into FBMN, the produced molecular networks with multiple information can clarify interesting compounds within intricate mixtures.

The “one-strain-many-compounds” (OSMAC) approach involves systematically varying culture conditions to explore the diversity of secondary metabolites in fungi, thereby facilitating the discovery of novel and bioactive compounds (Bode et al. 2002; Pan et al. 2019). In this study, we used the OSMAC approach to culture the marine algicolous fungus *T. harzianum* NTU2180, and then used FBMN, GNPS, and SIRIUS to provide an overview of metabolites from different fermentation methods. Utilizing the effective OSMAC approach and a feature-based molecular network analysis, the current study focused on the isolation and bioactivity evaluation of terpenoid-rich *T. harzianum* NTU2180 fermentation on germinated brown rice (GBR).

Retinoblastoma, a prototypical hereditary cancer, is an uncommon but severe childhood malignancy (Cruz-Gálvez et al. 2022). It originates from retinal cones, which possess specific properties that render them particularly prone to tumorigenesis. Despite its relatively low incidence, occurring in approximately 1 in every 18,000 live births on average, the condition can escalate to a life-threatening malignancy if not promptly treated (Byroju et al. 2023). The clinical management of retinoblastomas also presents formidable challenges, and prognosis tends to be unfavorable, especially in developing nations (Xiong et al. 2018). The progression of retinoblastomas is intricately tied to the angiogenic response, as evidenced by the presence of a substantial and diverse vasculature in retinoblastoma tumor samples. This vasculature comprises both neovascularization and mature vasculature with pericyte commitment, as determined through an immunohistochemical (IHC) analysis (Li et al. 2020). Blood vessels are crucial for embryonic development, growth, and wound healing. Additionally, neovascularization, a vital process for tumor growth and metastasis, facilitates the transportation of nutrients and elimination of metabolic wastes from tumor cells (Jiang et al. 2020). Therefore, meditating the tumor microenvironment by anti-angiogenesis is a dominant strategy for retinoblastoma treatment (Li et al. 2020). Mounting evidence supports that terpenoids demonstrate their anticancer activities through various molecular mechanisms, including anti-angiogenesis (Wróblewska-Łuczka et al. 2023). Here, we investigated the anti-angiogenesis potentials of secondary metabolites derived from terpenoid-rich *T. harzianum* NTU2180 fermentation on GBR for retinoblastoma treatment.

## Results

**Fig. 1:**
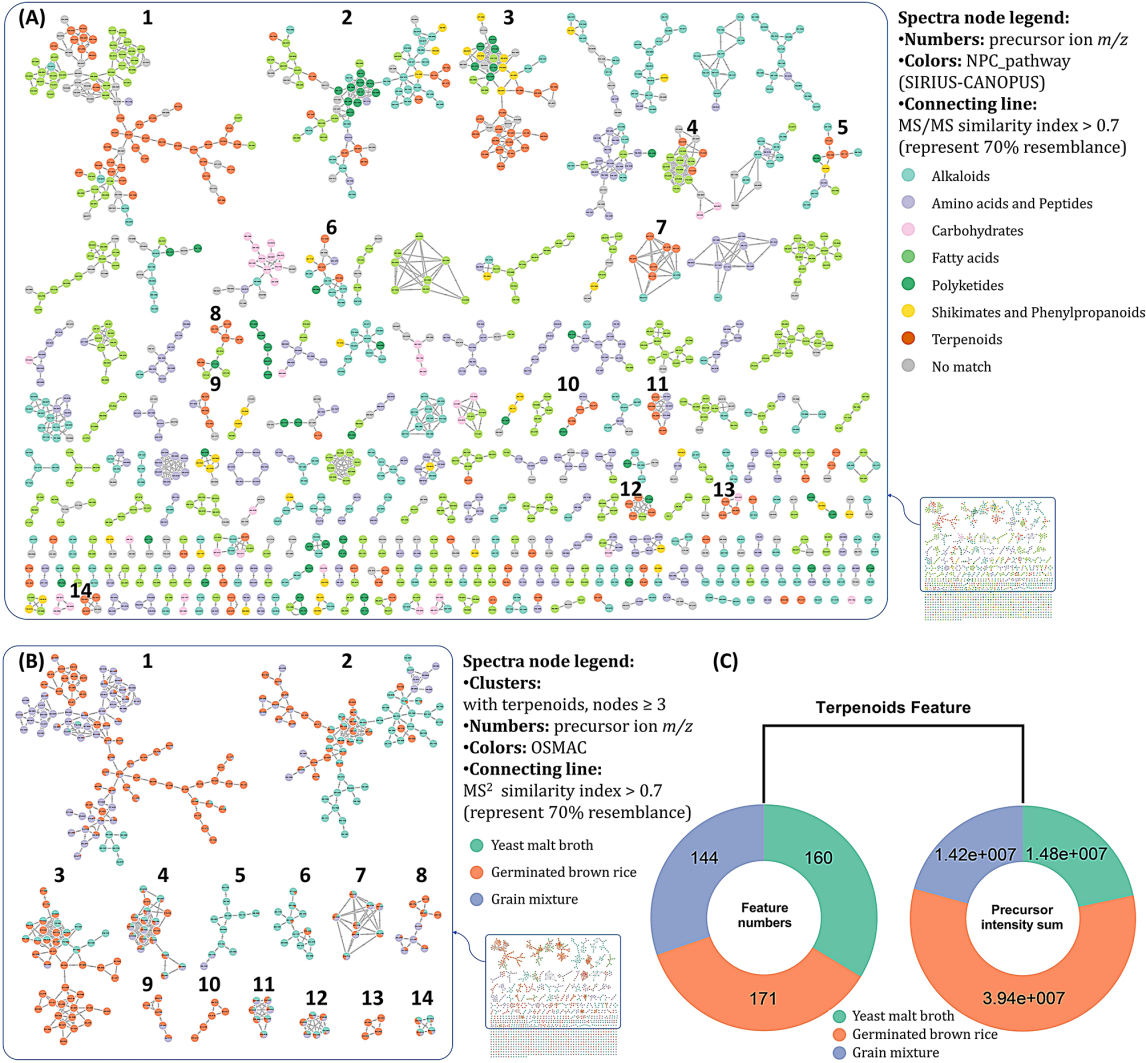
Feature-based molecular networking (FBMN) of the OSMAC approach to fermentation of *Trichoderma harzianum* NTU2180. (**A**) Painted by the classification of NPC_pathway from SIRIUS-CANOPUS software. Targeted molecular features containing terpenoids are in orange. (**B**) Clusters containing greater three or more terpenoid nodes were chosen to discuss the culture methods. (**C**) Feature numbers and precursor intensity calculations of all terpenoid nodes from (**A**)

Based on the OSMAC approach, three different fermentation methods of *T. harzianum* NTU2180 were used: MY broth, GBR, and GM. Crude extracts were obtained and analyzed by UHPLC-MS/MS, and chromatographic and spectrometric data were classified with NPClassifier (Kim et al. 2021) and annotated to construct a feature-based molecular network via the GNPS platform (Fig. 1A). In order to understand which fermentation methods of *T. harzianum* NTU2180 produced more terpenoids, clusters with three or more terpenoid nods were chosen and integrated with the three culture methods (Fig. 1B). Results showed that *T. harzianum* NTU2180 fermentation on GBR produced more terpenoids than the other two culture methods. Calculating the feature number and precursor intensity of terpenoids also obtained consistent results (Fig. 1C). Therefore, in the current study, *T. harzianum* NTU2180 fermentation on GBR was chosen to investigate terpenoid compounds and their anti-angiogenic activity.

**Fig. 2:**
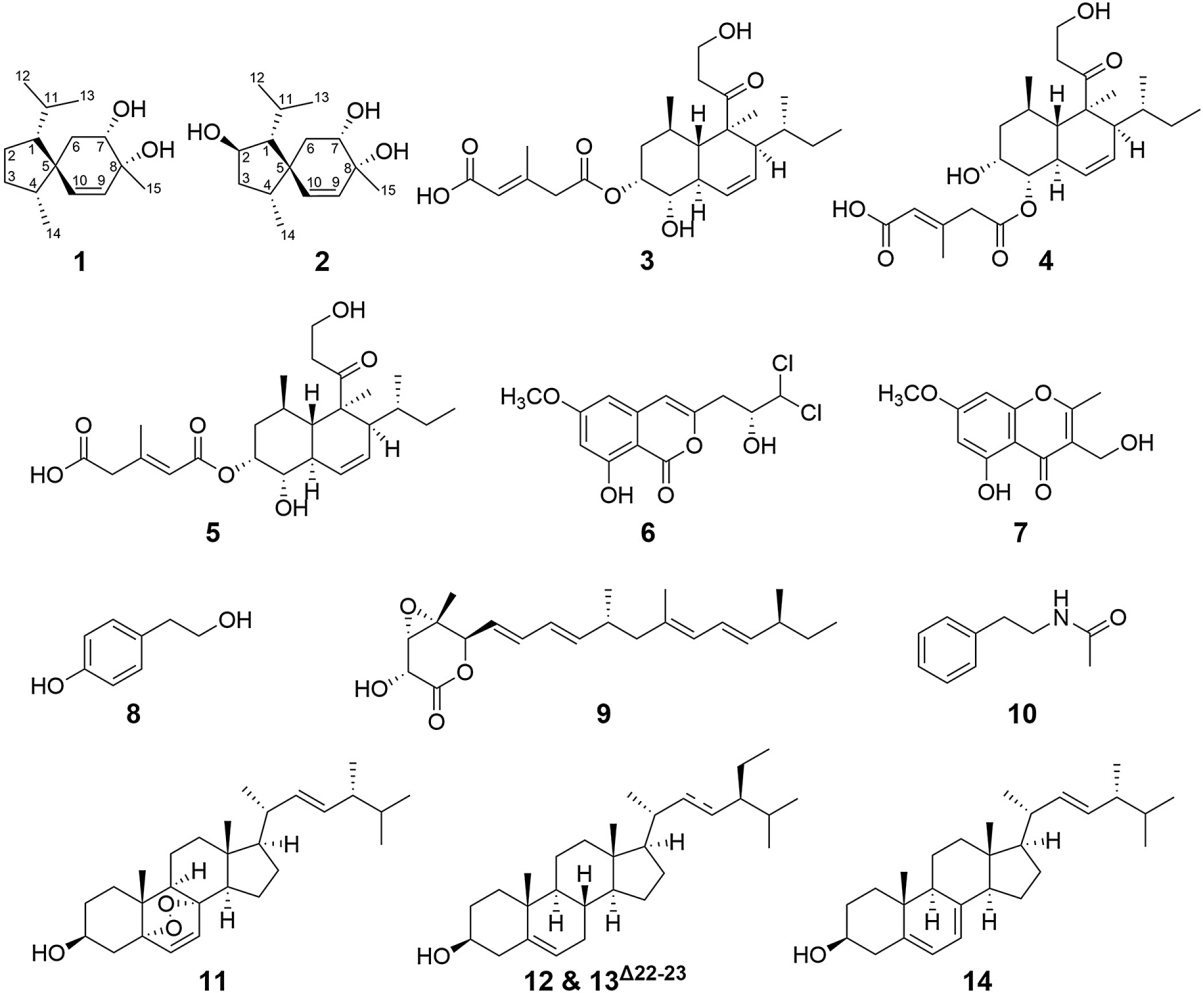
Structures of compounds **1** − **14**

The methanolic extract of *T. harzianum* NTU2180 fermentation on GBR was partitioned with EtOAc and water to obtain EtOAc-soluble and water-soluble layers. The EtOAc-soluble layer was chromatographed with a gradient solvent system to obtain 15 fractions. After overviewing FBMN of EtOAc fractions from *T. harzianum* NTU2180 fermentation on GBR, those fractions with terpenoids were targeted for further isolation and purification (see Supplementary information, Figure S2). Based on the guidance of MN and the OSMAC approach, we successfully isolated two new acorane-sesequiterpenes, trichospirols A (**1**) and B (**2**), and 12 known compounds (**3** − **14**) (Fig. 2). The proposed biosynthesis pathway of new compounds is discussed in the current study. In addition, eight compounds in sufficient amounts were evaluated for their anti-angiogenesis and anti-retinoblastoma activities.

**Fig. 3:**
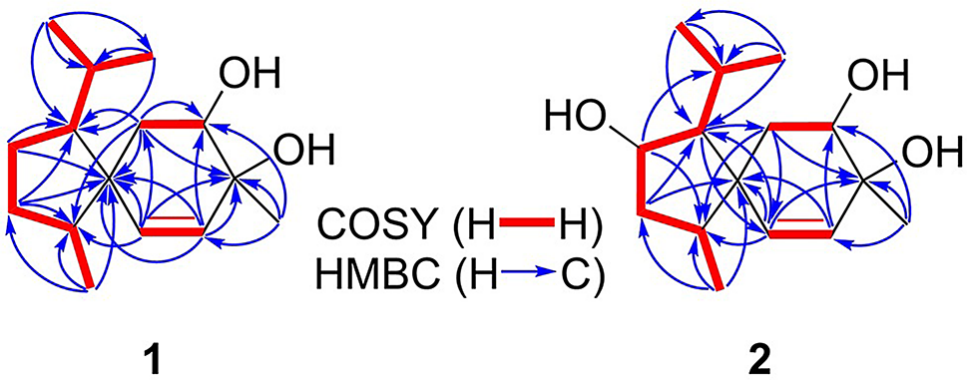
Key ^1^H-^1^H COSY and HMBC correlations of **1** and **2**

**Fig. 4:**
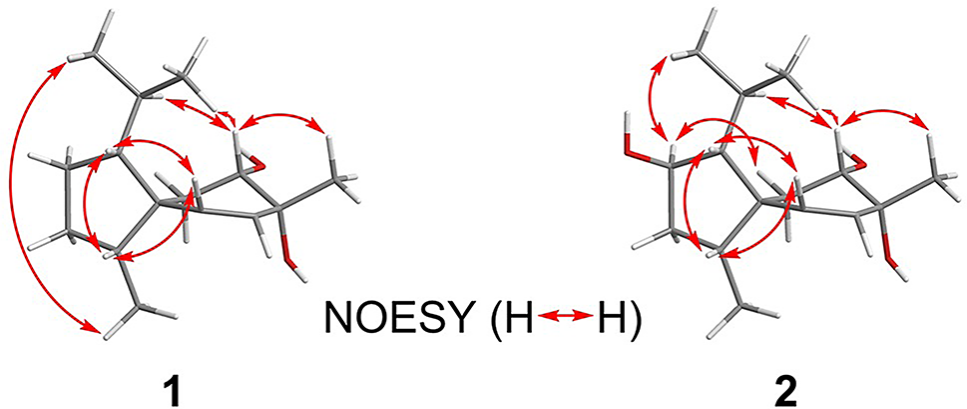
Stereochemical conclusions and key NOESY correlations of **1** and **2**

**Fig. 5:**
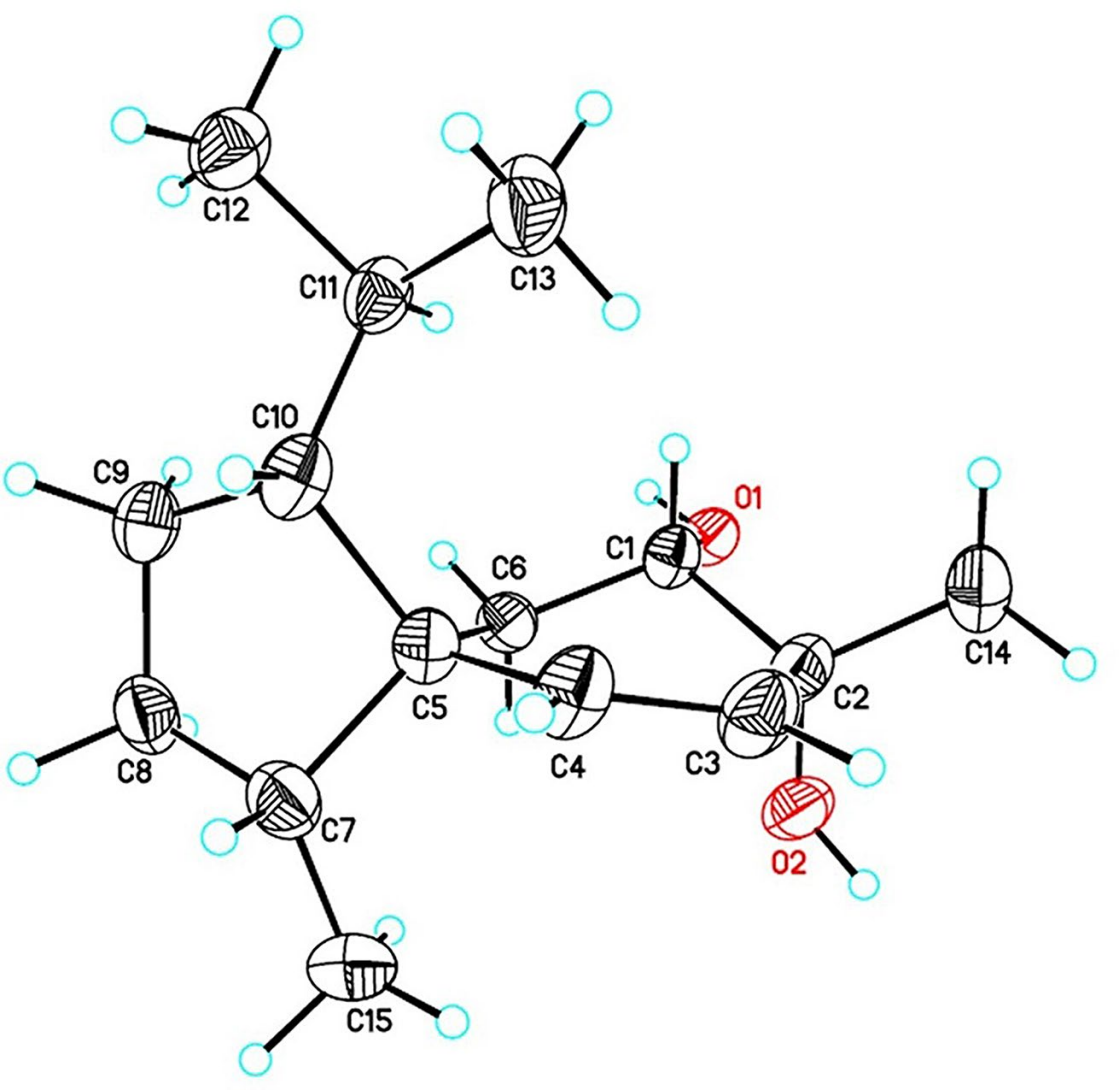
ORTEP drawing of **1** as determined by x-ray analyses. X-ray (Cu): C_15_H_26_O_2_, *M* = 238.36, crystal size: 0.20 × 0.02 × 0.01 mm^3^, crystal system: trigonal, space group: P3_2_, *a* = 12.7750(5) Å, *b* = 12.7750(5) Å, *c* = 15.6583(9) Å, α = β = 90°, γ = 120°, *V*: 2213.1(2) Å^3^, T = 100(2) K, *Z* = 6, d_calcd_ = 1.073 Mg/m3, λ(CuKα) = 1.54178 Å, *F*(000) = 792, reflections collected/independent reflections 37,778/5960 [R(int) = 0.0964], final *R* indices *R*_1_ = 0.0668 and *wR*_2_ = 0.1727, GOF on F^2^ = 1.050, Friedel pairs = 2964, absolute structure parameter = 0.36(18)

Compound **1** was obtained as optically colorless needles. The molecular formula of **1** was determined to be C_15_H_26_O_2_ through high-resolution electrospray ionization mass spectrometry (HRESIMS), indicating three indices of hydrogen deficiency (IHDs). The IR absorption of **1** revealed the presence of an -OH group (3336 cm^− 1^) and a C = C double bond (1646 cm^− 1^). The ^1^H NMR spectrum of **1** showed one singlet methyl group at δ_H_ 1.34 (s, H-15), three doublet methyl groups at δ_H_ 0.78 (d, *J* = 7.2 Hz, H-14), 0.85 (d, *J* = 6.6 Hz, H-12), and 0.87 (d, *J* = 6.6 Hz, H-13), six methylene signals at δ_H_ 1.14 (m, H-3b), 1.33 (m, H_ax_-6), 1.45 (brtd, *J* = 12.0, 3.6 Hz, H-2b), 1.51 (dd, *J* = 14.4, 4.8 Hz, H_eq_-6), 1.76 (m, H-3a), and 1.81 (m, H-2a), one oxymethine at δ_H_ 3.65 (dd, *J* = 12.6, 4.8 Hz, H_ax_-7), and a pair of double bonds at δ_H_ 5.36 (d, *J* = 9.6 Hz, H-10) and 5.68 (d, *J* = 9.6 Hz, H-9). (*Z*)-Double bond (C-9/C-10) was accordingly assigned based on the coupling constant of H-9/H-10 (*J* = 9.6 Hz) (Table 1). ^13^C NMR and HSQC data of **1** revealed 15 carbon signals attributable to four methyls [δ_C_ 13.8 (C-14), 22.9 (C-12), 23.5 (C-13), and 25.8 (C-15)], three methylenes [δ_C_ 26.6 (C-6), 27.3 (C-2), and 29.3 (C-3)], three methines [δ_C_ 30.2 (C-11), 46.5 (C-4), and 58.0 (C-1)], one oxymethine [δ_C_ 71.9 (C-7)], a double bond [δ_C_ 131.3 (C-9) and 141.6 (C-10)], one quaternary carbon [δ_C_ 50.5 (C-5)], and one oxygenated quaternary carbon [δ_C_ 68.6 (C-8)] (Table 1). The ^1^H-^1^H COSY plot of H-12/H-11/H-13 and H-11/H-1/H-2/H-3/H-4/H-14 revealed the existence of a 2-methylheptanyl group (Fig. 3). The HMBC correlation from H-2, H-3, and H-14 to C-5 indicated the presence of a five-member ring (C-1 to C-5)(Fig. 3). Further COSY correlation of H-6/H-7, H-9/H-10 and HMBC correlation of H-6/C-5 and C-10, H-9/C-5 and C-7, and H-10/C-5, C-6, and C-8 supported the existence of a six-membered ring (C-5 to C-10) (Fig. 3). The HMBC correlation from H-15 to C-7, C-8, and C-9 verified the junction between C-8 and C-15. Cross-peaks of H-6/C-1, C-4, and C-5, H-10/C-4 and C-5 in the HMBC spectrum proved that compound **1** is a spiro[4.5]decane derivative called acorane-type sesquiterpene. Finally, based on the chemical shifts of C-7 (δ_C_ 71.9) and C-8 (δ_C_ 68.6), the hydroxy groups were attached to C-7 and C-8, respectively. The NOESY cross-peaks of H-1/H-4, H-1/H-10, and H-4/H-10 indicated that H-1, H-4, and H-10 were all on the same face (Fig. 4). The large coupling constant of H_ax_-6/H-7 (*J* = 12.6 Hz) revealed that H-7 occupied an axial position. The key NOESY correlation of H-7/H-15 confirmed that the methyl group (C-15) occupied an equatorial position. The absolute configuration of **1** was determined by x-ray with Cu K-α radiation (Fig. 5). Accordingly, the structure of **1** was identified as (1*R*,4*R*,5*R*,7*S*,8*R*)-1-isopropyl-4,8-dimethylspiro[4.5]dec-9-ene-7,8-diol and named trichospirol A.

**Table 1:** ^1^H and ^13^C NMR data of compounds **1** and **2**

Position	1		2	
	δ_H_ mult. (J in Hz)	δ_C_	δ_H_ mult. (J in Hz)	δ_C_
1	1.39 m	58.0	1.32 m	66.7
2	1.45 brtd (12.0, 3.6)	27.3	4.18 m	75.0
	1.81 m			
3	1.14 m	29.3	1.14 m	41.4
	1.76 m		1.76 m	
4	1.63 m	46.5	1.98 m	43.4
5		50.5		51.9
6	1.33 m	26.6	1.33 t (12.7)	28.3
	1.51 dd (14.4, 4.8)		1.40 dd (12.7, 5.6)	
7	3.65 dd (12.6, 4.8)	71.9	3.63 dd (12.7, 5.6)	71.7
8		68.6		68.2
9	5.68 d (9.6)	131.3	5.70 d (9.9)	131.7
10	5.36 d (9.6)	141.6	5.41 d (9.9)	140.5
11	1.66 m	30.2	1.85 m	28.5
12	0.85 d (6.6)	22.9	1.06 d (7.2)	22.5
13	0.87 d (6.6)	23.5	0.94 d (6.6)	24.2
14	0.78 d (7.2)	13.8	0.80 d (6.6)	13.2
15	1.34 s	25.8	1.34 s	26.0

Data measured at 600 (^1^H) and 150 (^13^C) MHz in CDCl_3_

Compound **2** was isolated as an optical colorless solid. It had the molecular formula C_15_H_26_O_3_, and as suggested by HRESIMS, it had one oxygen unit more than that of compound **1**. By comparing the ^1^H and ^13^C NMR spectra of compound **2** with those of compound **1**, it was observed that the methylene group at C-2 in compound **1** was replaced by a hydroxy group in compound **2.** The change was confirmed by the chemical shift at C-2 (δ_C_ 75.0) and the molecular formula. Further the HMBC correlation between H-2/C-4 and C-11 verified the junction between hydroxy group and C-2 (Fig. 3). The cross-peak of H-2/H-12 in the NOESY spectrum established that the isopropyl group and H-2 were on the same face (Fig. 4). The remaining chiral centers of compound **2** were deduced to be of the 1*R*,4*R*,5*R*,7*S*,8*R*-form based on the same NMR patterns as compound **1**. Based on these assignments, the entire structure of compound **2** was deduced as shown (Fig. 2) and named trichospirol B.

The other 12 previously reported compounds were identified by comparing spectral data ([α]^X^_D_, UV, IR, NMR, and MS) to the literature, which included tandyukisin G (**3**) (Lai et al. 2022), tandyukisin H (**4**) (Lai et al. 2022), trichoharzin (**5**) (Kobayashi et al. 1993), dichlorodiaportin (**6**) (Larsen et al. 1999), 5-hydroxy-3-hydroxmethyl-2-methyl-7-methoxychromone (**7**) (Tanahashi et al. 2000), tyrosol (**8**) (Claydon et al. 1985), nafuredin (**9**) (Nagamitsu et al. 2008), N-phenethylacetamide (**10**) (Ding et al. 2023), ergosterol peroxide (**11**) (Gunatilaka et al. 1981), β-sitosterol (**12**) stigmasterol (**13**) (Kojima et al. 1990), and ergosterol (**14**) (Valisolalao et al. 1983). Furthermore, compounds **1**, **2**, **3**, **4**, **5**, and **11**, annotated on the FBMN of EtOAc layers of *T. harzianum* NTU2180 fermentation on GBR, belong to terpenoid classification by the NPClassifier (see Supplementary information, Figure S2).

**Scheme 1:**
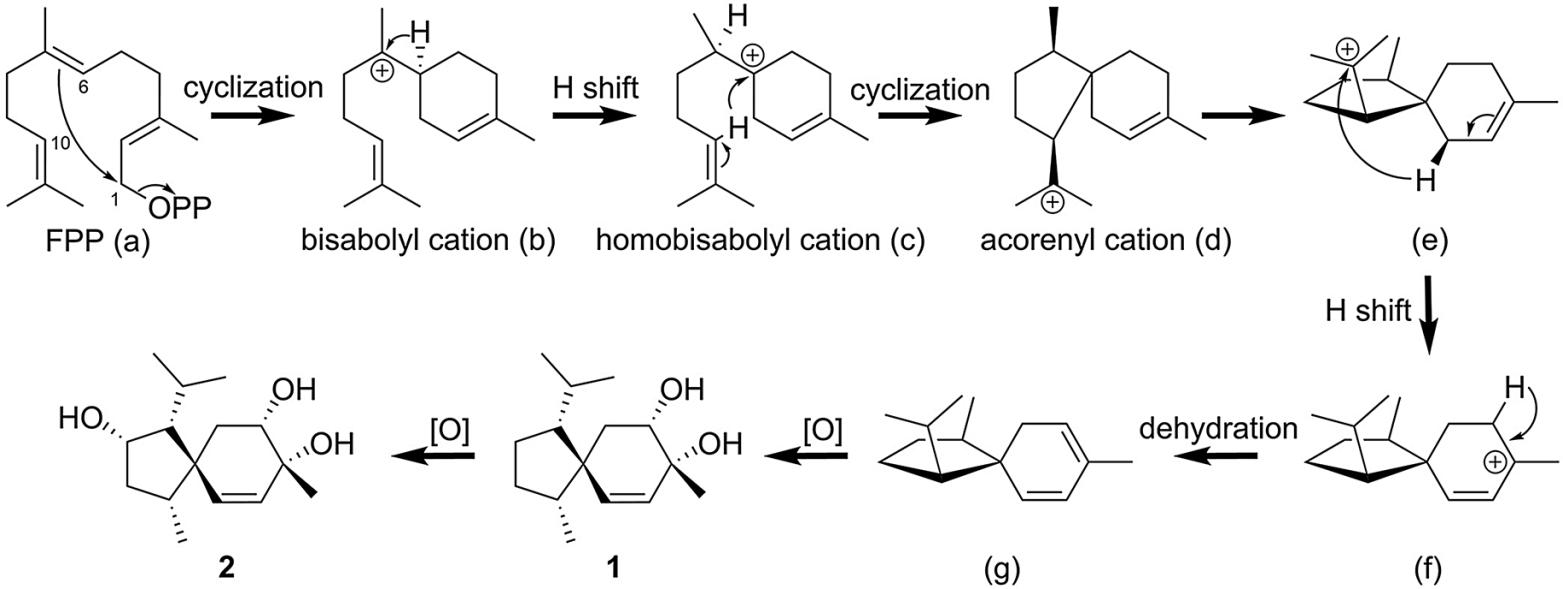
Proposed biosynthetic pathway of compounds **1** and **2**

**Fig. 6:**
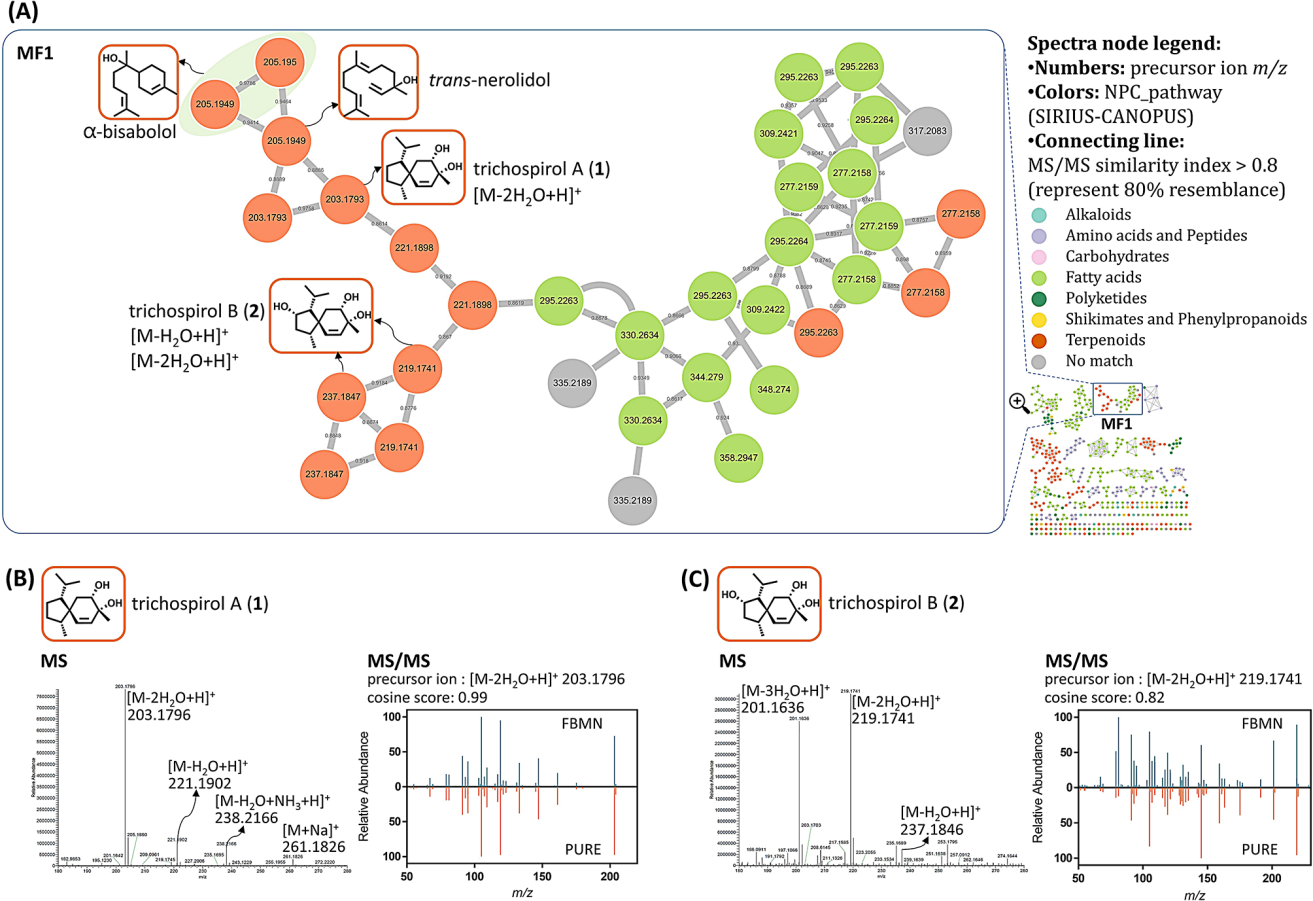
Feature-based molecular networking (FBMN) of EtOAc layers of *Trichoderma harzianum* NTU2180 fermentation on GBR and targeted molecular families (MFs) with new compounds. (**A**) The selected cluster **MF1** contained new compounds **1**, **2**, and their biosynthetic precursor-related compounds, α-bisabolol and trans-nerolidol. They were painted using classification of the NPC_pathway from SIRIUS-CANOPUS software. Values in the nodes are precursor ion m/z. (**B**) MS and MS/MS spectrum of compound **1**. The largest peak at *m/z* 203.1796 is the main precursor ion of [M-2H_2_O + H]^+^. (**C**) MS and MS/MS spectrum of compound **2**. The largest peak at *m/z* 219.1741 is the main precursor ion of [M-2H_2_O + H]^+^

Studies on the biosynthesis of acorane-type sesquiterpene suggested that they are formed by farnesyl pyrophosphate (FPP) (Yong et al. 2023). In a plausible biosynthetic pathway (Scheme 1), the FPP-initiated 1,6-cyclization could generate a bisabolyl cation (**b**). Then, the cation of (**b**) shifted to C-6 (**c**), and 6,10-cyclization formed an acorenyl cation (**d**). Subsequently, a beta-proton (H-10) shifted to C-11 in (**d**) to form intermediate (**e**), and intermediate (**e**) underwent a hydride shift to generate carbocation (**f**). The carbocation (**f**) underwent dehydration and oxidation, which led to the formation of **1** and **2**. Notably, the compounds related to biosynthetic precursors, α-bisabolol and *trans*-nerolidol, annotated by GNPS, were clustered with new compounds **1** and **2** (Fig. 6). This observation suggests a plausible biosynthetic hypothesis.

**Fig. 7:**
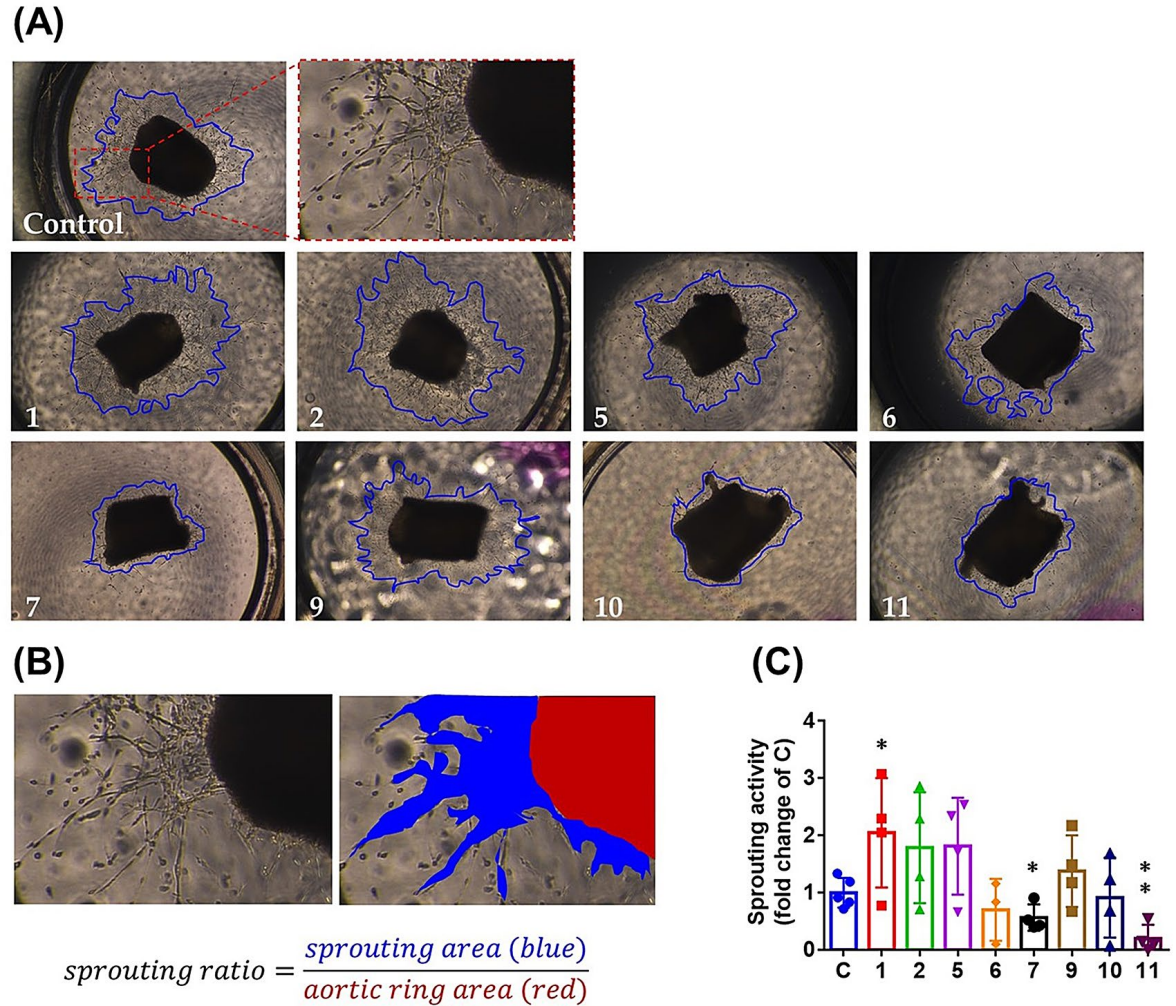
Ex vivo anti-angiogenesis effects of compounds by a sprouting assay. (**A**) Mouse aortic rings were embedded in Matrigel and treated with or without compounds. Representative images from day 7 of aortic rings and areas of vascular sprouting are framed with blue lines. A zoom of the sprouting area in the control group is shown in the inset. (**B**) The area occupied by vascular sprouting is marked blue, while the aorta explant is marked red. Sprouting activity was calculated as the area of vascular sprouting (blue color) / area of the aorta explant (red color). (**C**) Quantified fold change of the sprouting area was compared to the control group (*n* = 3 ~ 5). C, control. * *p* < 0.05, ** *p* < 0.005 compared to the control group

**Fig. 8:**
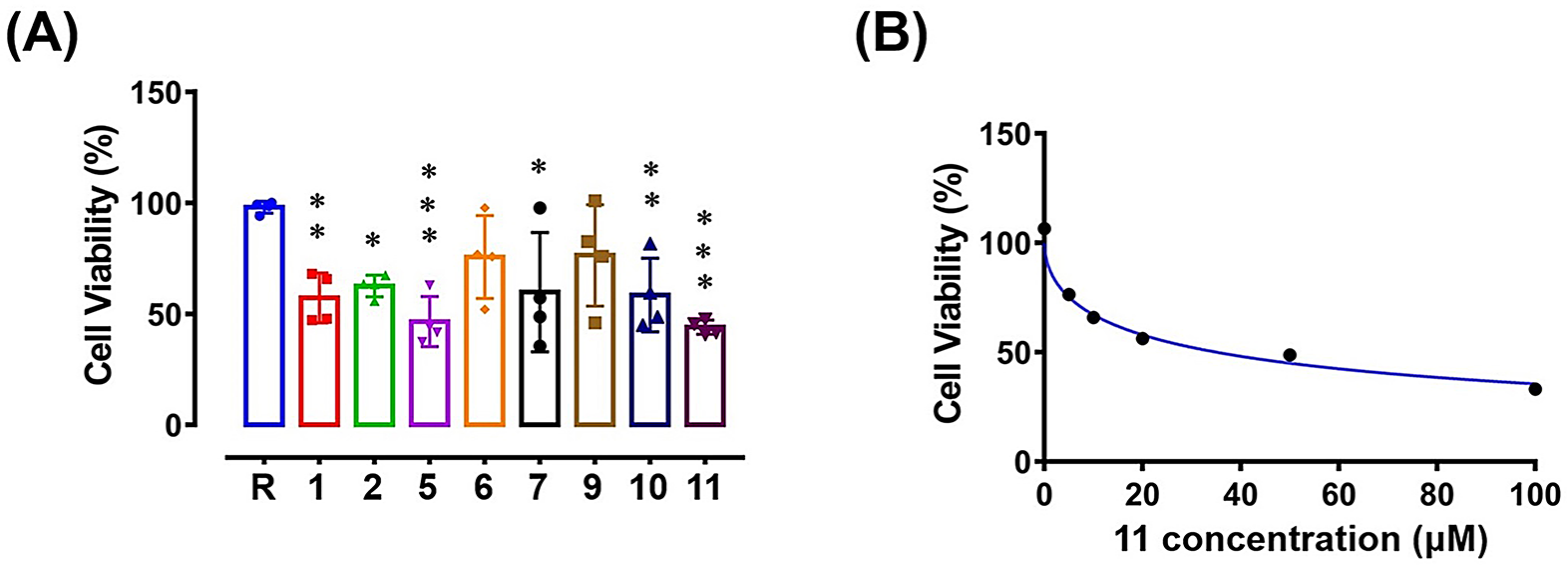
Effects of compounds on Y79 retinoblastoma cell growth. (**A**) After treatment with compounds for 48 h, human Y79 cell viability was tested by a colorimetric MTT assay. The absorbance of the compounds-treated group was conducted as a fold-change of the control resting group (*n* = 4). (**B**) The IC_50_ of compound **11** was 35.3 ± 6.9 µM (*n* = 5 ~ 6) R, resting. * *p* < 0.05, ** *p* < 0.005, *** *p* < 0.001 compared to the resting group

We explored the anti-angiogenesis effects of eight compounds (compounds **1**, **2**, **5** − **7**, and **9** − **11**), which were derived in sufficient amounts, in ex vivo thoracic aorta explants isolated from wild-type C57BL/6 mice. Newly formed vascular sprouting (framed with a blue line) representing angiogenesis varied after incubation of aortic rings with compounds (20 µM) (Fig. 7A). Sprouting activity was calculated as the area of vascular sprouting (blue color) / area of aorta explant (red color) rather than the sprouting area alone to avoid tissue sample size-related bias (Fig. 7B). 5-Hydroxy-3-hydroxmethyl-2-methyl-7-methoxychromone (**7**) (0.57 ± 0.12-fold) and ergosterol peroxide (**11**) (0.20 ± 0.12-fold) treatment significantly reduced angiogenesis compared to the control (1.00 ± 0.11-fold) at observation time point day 7 (Fig. 7C). In contrast, trichospirol A (**1**) enhanced angiogenesis (2.05 ± 0.48-fold). To test the antitumor effect of isolates, Y79 retinoblastoma cells were exposed to eight compounds (compounds **1**, **2**, **5** − **7**, and **9** − **11**) at a concentration of 20 µM for 48 h, and their cell viability was evaluated by an MTT assay. After 48 h of incubation, ergosterol peroxide (**11**) (0.75 ± 0.03-fold) alone significantly suppressed Y79 proliferation compared to the control group (1.00 ± 0.13-fold) (Fig. 8A). The results shown in Fig. 8B indicated that compound **11** inhibited Y79 retinoblastoma cell growth in a concentration-dependent manner with an IC_50_ value of 35.3 ± 6.9 µM.

## Discussion

In the current study, employing the OSMAC approach and MN guidance resulted in the discovery of two new compounds along with 12 known compounds from GBR fermentation of *T. harzianum* NTU2180. Among them, trichospirols A (**1**) and B (**2**) are acorane-type sesquiterpenes generated by a spiro[4.5]decane with an isopropyl unit and dimethyl substitution. Acorane-type sesquiterpenes can only be found in some plants, including of the Chloranthaceae (Yan et al. 2024), Schisandraceae (Sun et al. 2023; Yong et al. 2023), Fabaceae (Han et al. 2022), and Euphorbia (Zhu et al. 2021). Notably, acorane-type sesquiterpenes have also been discovered in some fungi, including Basidiomycota (Sum et al. 2023), *Trichoderma* sp. (Wang et al. 2022a, b), and *Penicillin* sp. (Zhang et al. 2022), and in a marine sponge, *Myrmekioderma* sp. (Zhou et al. 2022). As a result of the distinctive skeleton, acroranes demonstrate a broad spectrum of bioactivities (Zhang et al. 2022). To our knowledge, this study marks the initial discovery of the inhibitory effects of an acroran-type sesquiterpene on ex vivo angiogenesis by an aorta ring sprouting assay. On the other hand, further research is needed to demonstrate whether the enhancement of angiogenesis by trichospirol A (**1**) can be applied for cell repair or wound healing. Moreover, our results from ex vivo and in vitro models respectively revealed the anti-angiogenesis potential and proliferation inhibitory activity of retinoblastomas by *T. harzianum* NTU2180. In particular, ergosterol peroxide (**11**) was identified as a dual inhibitor targeting both angiogenesis and retinoblastoma proliferation, and 5-hydroxy-3-hydroxmethyl-2-methyl-7-methoxychromone (**7**) was mainly involved in anti-angiogenesis, which can potentially be developed for angiogenesis-related ocular diseases such as diabetic retinopathy and corneal angiogenesis.

## Conclusions

MS/MS analysis of various fermentation methods contributes valuable insights for optimizing fermentation conditions to obtain specific skeleton compounds. With FBMN strategy, terpenoids were identified as the major NPC class in the GBR fermentation of *T. harzianum* NTU2180 in the current study. The FBMN-guided fractionation identified two new acorane-sesquiterpenes, trichospirols A (**1**) and B (**2**), and fourteen components **1**–**14**. The proposed biosynthetic pathway was also revealed. The anticancer activity of isolates was revealed by ex vivo anti-angiogenesis experiment and in vitro proliferation inhibitory activity of retinoblastomas test, respectively. Taken together, findings from this study unveiled the chemical characteristics and bioactivities of *T. harzianum* NTU2180, providing solid evidence for the development of *T. harzianum* on retinoblastoma treatments.

## Methods

### General experimental procedures

^1^H, ^13^C, 1D, and 2D nuclear magnetic resonance (NMR) spectra were acquired on an Agilent DD2 600 MHz NMR (Agilent, CA, USA) and Bruker AVIII 800 MHz NMR with Cryoprobe (Bruker, Germany). High-resolution mass spectrometric (MS) and tandem MS (MS/MS) spectra were obtained using Thermo DIONEX UltiMate 3000 ultra-high-performance liquid chromatography (UHPLC) coupled to a Thermo Q-Exactive Focus Orbitrap mass spectrometer system (Thermo, MA, USA) and Thermo DIONEX UltiMate 3000 UHPLC coupled to a Thermo Q-Exactive Plus Orbitrap mass spectrometer system (Thermo). Optical rotations and ultraviolet (UV) spectra were respectively measured on a Jasco P-2000 polarimeter (Jasco, Japan) and a Thermo UV-Visible Helios α spectrophotometer (Bellefonte, CA, USA). Infrared (IR) spectra were recorded on a Jasco FT/IR 4100 spectrometer (Jasco). TLC was performed using silica gel 60 F_254_ plates and silica gel 60 RP-18 F_254_ plates with a thickness of 0.2 mm from Merck (Germany). Flash medium-performance liquid chromatography (Flash-MPLC) was performed using Santai Sepabean™ machine 2 (Santai, China) with silica gel (SiliaFlash^®^ P60, 230–400 mesh; Silicycle, Canada). MPLC was carried out with glass columns packed with silica gel (Chromatorex SO_3_H MB100-40/75 and SMB 100 − 20/45, Fuji Silysia Chemical, Japan) and RP-C18 (YMC ODS-A-HG, 12 nm, S-50, MA, USA), performed using a Waters 515 HPLC Pump (Waters, MA, USA). Sephadex LH-20 from Amersham Biosciences (Amersham Biosciences, Sweden) was used for open column chromatography. Further purification steps were performed by high-performance liquid chromatography (HPLC) using the Shimadzu SCL-40 system with an SPD-M40 photodiode array (PDA) detector (Shimadzu, Japan).

### Identification and OSMAC approach to fermentation of Trichoderma Harzianum NTU2180

*Trichoderma harzianum* NTU2180 was isolated from *Ulva intestinalis* Linn. collected from the northeast coast of Taiwan. The strain was identified by Prof. Tzong-Huei Lee and deposited at the Institute of Fisheries Science, National Taiwan University (No. NTU2180). The internal transcribed spacer (ITS) gene sequence was deposited in NCBI’s GenBank with accession no. BU1MVK80013. Before OSMAC fermentation, the strain was inoculated with sterilized malt yeast (MY) broth (2.4 g of malt extract and 0.6 g of yeast extract (from Becton Dickinson, MD, USA) in 100 mL H_2_O), conducted at 25–30 °C for 2 days in 250-mL flasks with constant shaking (180 rpm). Then 20 mL was taken to inoculate each OSMAC culture. Three different fermentation conditions were employed for *T. harzianum* NTU2180 as follows: MY: 100 mL MY broth in 250-mL flasks, with constant shaking at 180 rpm for 10 days; germinated brown rice (GBR): 100 g GBR with 35 mL MY broth and 35 mL H_2_O, allowed to stand for 20 days; and grain mixture (GM): 100 g GM with 35 mL MY broth and 35 mL H_2_O, allowed to stand for 20 days. For large-scale fermentation, a mixture of 600 g GBR, 180 mL MY broth, and 180 mL H_2_O was sterilized and then introduced into glass culture flasks, where the fermentation process continued for 40 days.

### Extraction and isolation

The product of MY fermentation of *T. harzianum* NTU2180 was filtered and extracted with an equal volume of EtOAc three times. The product of solid-state fermentation (GBR and GM) of *T. harzianum* NTU2180 was extracted with methanol three times at room temperature. The methanolic syrup extract was partitioned with EtOAc and H_2_O (1:1) to give EtOAc-soluble and H_2_O-soluble layers.

The product of large-scale fermentation of *T. harzianum* NTU2180 on GBR was extracted with MeOH three times at room temperature. The methanolic syrup extract (62.0 g) was partitioned with EtOAc and H_2_O (1:1) to give an EtOAc-soluble layer (15.2 g). The EtOAc-soluble layer was subjected to Flash-MPLC (silica gel; 100% *n*-hexane to 100% acetone, then washed with 100% methanol) to produce 15 fractions (Fr. 1 ~ 15).

Fraction (Fr.) 6 was subjected to MPLC (silica gel; *n*-hexane/acetone 5:1) to obtain nine subfractions, which contained a mixture of **12** with **13** (4.25 mg), and **14** (29.75 mg). Fr. 8 was subjected to MPLC (RP-C18 gel; water/methanol 1:4) to obtain five subfractions, including **1** (2.45 mg). Compound **1** was crystallized using methanol to give optically colorless needles. Fr. 10 was applied to MPLC (RP-18; water/methanol 1:15) to give ten subfractions and obtain **11** (17.32 mg). Fr. 10 − 1 and 10 − 2 were combined and eluted by MPLC (silica gel; *n*-hexane/EtOAc 4:1) to obtain **6** (3.93 mg). Fr. 10 − 3 was purified by thin-layer chromatography (TLC) using dichloromethane/acetone (20:1, v/v) for development to yield **9** (*R*_*f*_ = 0.68; 1.01 mg). Fr. 11 was subjected to MPLC (RP-18; water/acetonitrile 1:2 with 0.1% formic acid) to obtain seven subfractions. Fr. 11 − 1 was purified by semi-prepared HPLC on an RP-18 column (60% MeOH, 0.1% formic acid, flow = 2 mL/min) to give **7** (*R*_*t*_ = 27.5 min; 3.85 mg). Fr. 12 was subjected to MPLC (RP-18; water/acetone 4:5) to obtain 21 subfractions. Fr. 12 − 3 was treated with semi-prepared HPLC on an RP-C18 column (40% MeOH, flow = 2 mL/min) to give **8** (*R*_*t*_ = 12.7 min; 0.10 mg). Fr. 12 − 4 was purified by semi-prepared HPLC on an RP-C18 column (45% MeOH, flow = 2 mL/min) to give **10** (*R*_*t*_ = 27.1 min; 4.14 mg) and **2** (*R*_*t*_ = 32.2 min; 1.76 mg). Fr. 13 was re-dissolved in 5 mL MeOH, applied to a Sephadex LH-20 column (2.0 cm i.d. × 75.0 cm), and eluted with MeOH to obtain nine fractions. Fr. 13 − 2 was subjected to MPLC (RP-C18 gel; water/acetone 1:1) to obtain 15 subfractions. Fr. 13-2-2 was purified by semi-prepared HPLC on an RP-C18 column (60% MeOH, flow = 2 mL/min) to give **3** (*R*_*t*_ = 28.3 min; 1.04 mg), **5** (*R*_*t*_ = 31.5 min; 5.50 mg), and **4** (*R*_*t*_ = 35.9 min; 0.46 mg). The isolation flow chart of large-scale fermentation of *T. harzianum* NTU2180 on GBR was shown in Supplementary information, Figure S1.

### Spectral data of new compounds 1 and 2

Trichospirol A (**1**): CCDC 2297704; optically colorless needles (methanol); [α]^28^_D_: +46.2 (*c* 0.1 MeOH); IR (ATR) *ν*_max_: 3336 (-OH), 1646 (C = C) cm^− 1^; ^1^H NMR (CDCl_3_, 600 MHz) and ^13^C NMR data (CDCl_3_, 150 MHz): see Table 1; HRESIMS [M + Na]^+^ at *m/z* 261.1825 (calcd. 261.1825 for C_15_H_26_O_2_Na^+^).

Trichospirol B (**2**): optically colorless solid; [α^28^_D_: +15.8 (*c* 0.1 MeOH); IR (ATR) *ν*_max_: 3379 (-OH) cm^− 1^; ^1^H NMR (CDCl_3_, 600 MHz) and ^13^C NMR data (CDCl_3_, 150 MHz): see Table 1; HRESIMS [M + H − 2H_2_O]^+^ at *m/z* 219.1741 (calcd. 219.1743 for C_15_H_23_O^+^).

### MN process

#### Non-targeted fragment ions collected by UHPLC-MS/MS

MS/MS data were collected from Thermo DIONEX UltiMate 3000 UHPLC coupled to a Thermo Q-Exactive Focus Orbitrap mass spectrometer spectrometric system. UHPLC separation was performed using a Thermo Hypersil GOLD™ C18 column (1.9 μm, 2.1 mm × 100 mm) (Fig. 1) and Waters Acquity UPLC^®^ HSS T3 column (1.8 μm, 2.1 mm × 100 mm) (Fig. 6). The mobile phase was set to A (water, containing 0.1% formic acid and 2 mM ammonium formate)/B (95% acetonitrile, 5% water, containing 0.1% formic acid and 2 mM ammonium formate), gradient sequences: 0–10 min, 5–100% B; 10–15 min, 100% B; 15–15.5 min, 100–5% B; 15.5–20 min, 5% B. The flow rate was set to 0.3 mL/min, and the oven temperature was maintained at 40 °C. Samples were dissolved in methanol to a concentration of 5000 ppm and then filtered through 0.2-µm PTFE syringe filter (Pall, NY, USA). Samples were automatically injected at 1 µL/injection. MS and MS/MS data were collected within the range of *m/z* 133.4–1500, and the top three precursor ions were fragmented with ramping of energies of 10, 20, and 40 eV.

#### Data preprocessing by MZmine3

The collected MS and MS/MS data were converted by MSConvert software to mzML format (Chambers et al. 2012), and then processed by MZmine software (vers. 3.9.0) (Schmid et al. 2023). The chromatogram was built with the Automated Data Analysis Pipeline (ADAP) chromatogram builder (Myers et al. 2017). The processed results were exported by “Export feature list” for an FBMN (Nothias et al. 2020) analysis in the Global Natural Products Social Molecular Networking (GNPS) (Wang et al. 2016) environment and for the software SIRIUS (Duhrkop et al. 2019). The similarity between FBMN and isolated compounds was assessed using cosine similarity (Florian et al. 2020).

#### FBMN in the GNPS environment

The GNPS web-based platform (Wang et al. 2016) was applied to analyze output data from MZmine3 to generate MS/MS MN (job ID: 25a9d4ae142742ed9af1cc763c0d1cee) (Fig. 1) and job ID: 776df4ef21e641a09b05264207522f03 (Fig. 6) with FBMN workflow (vers. 28.2). The network was created in which linkages between nodes were filtered by cosine scores above 0.7 and 0.8, with at least six matched fragment ions. The FBMN was visualized and laid out using Cytoscape 3.10.1 software (Shannon et al. 2003) with yFiles Organic Layout, supplied by “yFiles Layout Algorithms” at the Cytoscape.

#### Data analysis, classification, and prediction by SIRIUS and CANOPUS

SIRIUS software was used to analyze metabolites from the featured MS/MS data output from MZmine3. Processing consisted of four parts: SIRIUS (Bocker et al. 2016; Bocker et al. 2009; Duhrkop et al. 2019)–molecular formula identification; ZODIAC (Ludwig et al. 2019)–network-based improvement of the SIRIUS molecular formula ranking; CSI: FingerID (Duhrkop et al. 2015; Hoffmann et al. 2021)–fingerprint prediction and structure database search; and CANOPUS (Djoumbou Feunang et al. 2016; Duhrkop et al. 2021; Kim et al. 2021)–compound class prediction. Results were exported by “Summaries”, and input into the visualized FBMN generated by GNPS in Cytoscape software (Shannon et al. 2003).

### Aortic ring sprouting assays

Aortic ring sprouting assays were conducted as previously described with some modifications (Wang et al. 2022a, b). Thoracic aortas were collected from euthanized C57BL/6 mice. After removing excess perivascular tissue, the aorta was transversely sectioned (~ 1 mm in width) and washed in endothelial cell medium (ECM) (1001; ScienCell, CA, USA). Each aortic ring was embedded in 50 µL Matrigel™ Basement Membrane Matrix (356234; Corning, NY, USA) in a 96-well plate, with the lumen parallel to the base. Once Matrigel had solidified, 100 µL of complete medium (ECM supplemented with antibiotics and serum) with or without compounds (20 µM) was added to each well, and the plate was incubated at 37 °C for 7 days. The medium was changed every 3 days until the experiment ended. The newly formed vascular sprouting area and aorta explant area were quantified by ImageJ software vers. 1.48 and calculated as sprouting activity.

### Cytotoxicity assay

The antitumor activity of compounds was evaluated using Y79 human retinoblastoma cells (6042; BCRC). Cell viability was assessed using a 3-(4,5-dimethylthiazol-2-yl)-2,5-diphenyltetrazolium bromide (MTT) assay. Y79 cells (10^4^) were resuspended in 100 µL RPMI medium with or without different compounds (20 µM) in a 96-well plate and incubated at 37 °C. After 48 h of incubation, 20 µL of the MTT reagent (5 mg/mL) was added to each well and incubated for 1.5 h. The water-insoluble formazan crystals formed in active mitochondria were dissolved in 50 µL of DMSO, and the absorbance at a wavelength of 595 nm was recorded on a microplate reader. The fold change in cell viability was determined by comparing it to the resting Y79 group.

## Electronic supplementary material

Below is the link to the electronic supplementary material.

Supplementary Material 1

## Data Availability

Data from this study is available from the corresponding author, Wu HC.

## Abbreviations

ADAP: Automated Data Analysis Pipeline
COSY: Correlation Spectroscopy
DEPT: Distortionless Enhancement by Polarization Transfer
ECM: Endothelial Cell Medium
FBMN: Feature-Based Molecular Networking
Flash-MPLC: Flash Medium- Pressure Liquid Chromatography
FPP: Farnesyl Pyrophosphate
GBR: Germinated Brown Rice
GM: Grain Mixture
GNPS: Global Natural Products Social Molecular Networking
HMBC: Heteronuclear Multiple Bond Correlation
HPLC: High-Performance Liquid Chromatography
HRESIMS: High-Resolution Electronic Spray Ionization Mass Spectrometry
IHC: Immunohistochemical
IHDs: Indicating Three Indices of Hydrogen Deficiency
IR: Infrared
ITS: Internal Transcribed Spacer
MFs: Molecular Families
MN: Molecular Networking
MPLC: Medium-Performance Liquid Chromatography
MS: Mass Spectrometry
MS/MS: Tandem Mass Spectrometer
MTT: 3-(4,5-dimethylthiazol-2-yl)-2,5-diphenyltetrazolium bromide
MY: Malt Yeast
NMR: Nuclear Magnetic Resonance
NOESY: Nuclear Overhauser Effect Spectroscopy
NPs: Natural Products
OSMAC: One-Strain-Many-Compounds
PDA: Photodiode Array
RPMI: Roswell Park Memorial Institute
*T. harzianum*: *Trichoderma harzianum*
TLC: Thin Layer Chromatography
UHPLC: Ultra-High-Performance Liquid Chromatography
UHPLC-MS/MS: Ultra High-Performance Liquid Chromatography–Tandem Mass Spectrometry
UV: Ultraviolet
