# Supplementary material for: New acorane-sesequiterpenes and anti-retinoblastoma constituents from the marine algicolous fungus Trichoderma harzianum NTU2180 guided by molecular networking strategy
Source: Bot Stud. 2025 Jan 14;66:2. doi: 10.1186/s40529-024-00449-5 (PMC11732828; doi:10.1186/s40529-024-00449-5)
Supplement: Supplementary file 1 — Supplementary Material 1 [file 40529_2024_449_MOESM1_ESM.docx]

Supplementary Material

**New acorane-sesequiterpenes and anti-retinoblastoma constituents from the marine algicolous fungus *Trichoderma harzianum* NTU2180 guided by molecular networking strategy**

Andrea Gu ^a,b^, Fan-Li Lin ^c,d^, Chung-Kuang Lu ^e^, Tz-Wei Yeh ^a,e^, Yih-Fung Chen ^f^, Ho-Cheng Wu ^b,g*^, Tzong-Huei Lee ^a*^

^a^ *Institute of Fisheries Science, College of Life Science, National Taiwan University, Taipei 106319, Taiwan*

^b^ *School of Pharmacy, College of Pharmacy, Kaohsiung Medical University, Kaohsiung 807378, Taiwan*

^c^ *Department of Pharmacology, School of Post-Baccalaureate Medicine, College of Medicine, Kaohsiung Medical University, Kaohsiung 807378, Taiwan*

^d^ *Department of Medical Research, Kaohsiung Medical University Hospital, Kaohsiung 807378, Taiwan*

^e^ *National Research Institute of Chinese Medicine, Ministry of Health and Welfare, Taipei 112304, Taiwan*

^f^ *Graduate Institute of Natural Products, College of Pharmacy, Kaohsiung Medical University, Kaohsiung 807378, Taiwan*

^g^ *Department of Medical Research, Kaohsiung Medical University Hospital, Kaohsiung 807378, Taiwan*

***Correspondence:**

Dr. Ho-Cheng Wu

School of Pharmacy, College of Pharmacy, Kaohsiung Medical University, Kaohsiung 807378, Taiwan
No. 100, Shiquan 1st Rd., Sanmin Dist., Kaohsiung 807378, Taiwan (R.O.C.)
Phone: +886 7 3121101 ext. 2672

***** E-mail: hcwu@kmu.edu.tw

Prof. Tzong-Huei Lee

Institute of Fisheries Science, College of Life Science, National Taiwan University, Taipei 106319, Taiwan
No. 1, Sec. 4, Roosevelt Rd., Da'an Dist., Taipei 106319, Taiwan (R.O.C.)

Phone: +886 2 33661828

***** E-mail: dthlee1@ntu.edu.tw**Content**

[**Figure S1.** The isolation flow chart of large-scale fermentation of *T. harzianum* NTU2180 on GBR. 1](#_Toc173920502)

[**Figure S2.** Feature-based molecular networking (FBMN) of the EtOAc fractions from *T. harzianum* NTU2180 fermentation on GBR. 2](#_Toc173920503)

[**Figure S3.** ^1^H NMR spectrum of compound **1** (CDCl_3_, 600 MHz) 3](#_Toc173920504)

[**Figure S4.** ^13^C NMR spectrum of compound **1** (CDCl_3_, 150 MHz) 3](#_Toc173920505)

[**Figure S5.** HSQC spectrum of compound **1** 4](#_Toc173920506)

[**Figure S6.** COSY spectrum of compound **1** 4](#_Toc173920507)

[**Figure S7.** HMBC spectrum of compound **1** 5](#_Toc173920508)

[**Figure S8.** NOESY spectrum of compound **1** 5](#_Toc173920509)

[**Figure S9.** HRESIMS spectrum of compound **1** 6](#_Toc173920510)

[**Figure S10.** ^1^H NMR spectrum of compound **2** (CDCl_3_, 600 MHz) 6](#_Toc173920511)

[**Figure S11.** ^13^C NMR spectrum of compound **2** (CDCl_3_, 150 MHz) 7](#_Toc173920512)

[**Figure S12.** HSQC spectrum of compound **2** 7](#_Toc173920513)

[**Figure S13.** COSY spectrum of compound **2** 8](#_Toc173920514)

[**Figure S14.** HMBC spectrum of compound **2** 8](#_Toc173920515)

[**Figure S15.** NOESY spectrum of compound **2** 9](#_Toc173920516)

[**Figure S16.** HRESIMS spectrum of compound **2** 9](#_Toc173920517)

**Figure S1.** The isolation flow chart of large-scale fermentation of *T. harzianum* NTU2180 on GBR.

**

Figure S2.** Feature-based molecular networking (FBMN) of the EtOAc fractions from *T. harzianum* NTU2180 fermentation on GBR.

(**A**) The three targeted molecular families (**MF1**, **MF2**, **MF3**) containing terpenoids for further isolation and purification were is framed in blue. (**B**) Painted by the EtOAc fractions of three MF clusters from (**A**). The isolated pure compounds were denoted by circles inside the MF clusters, and annotated with structure and precursor ion.


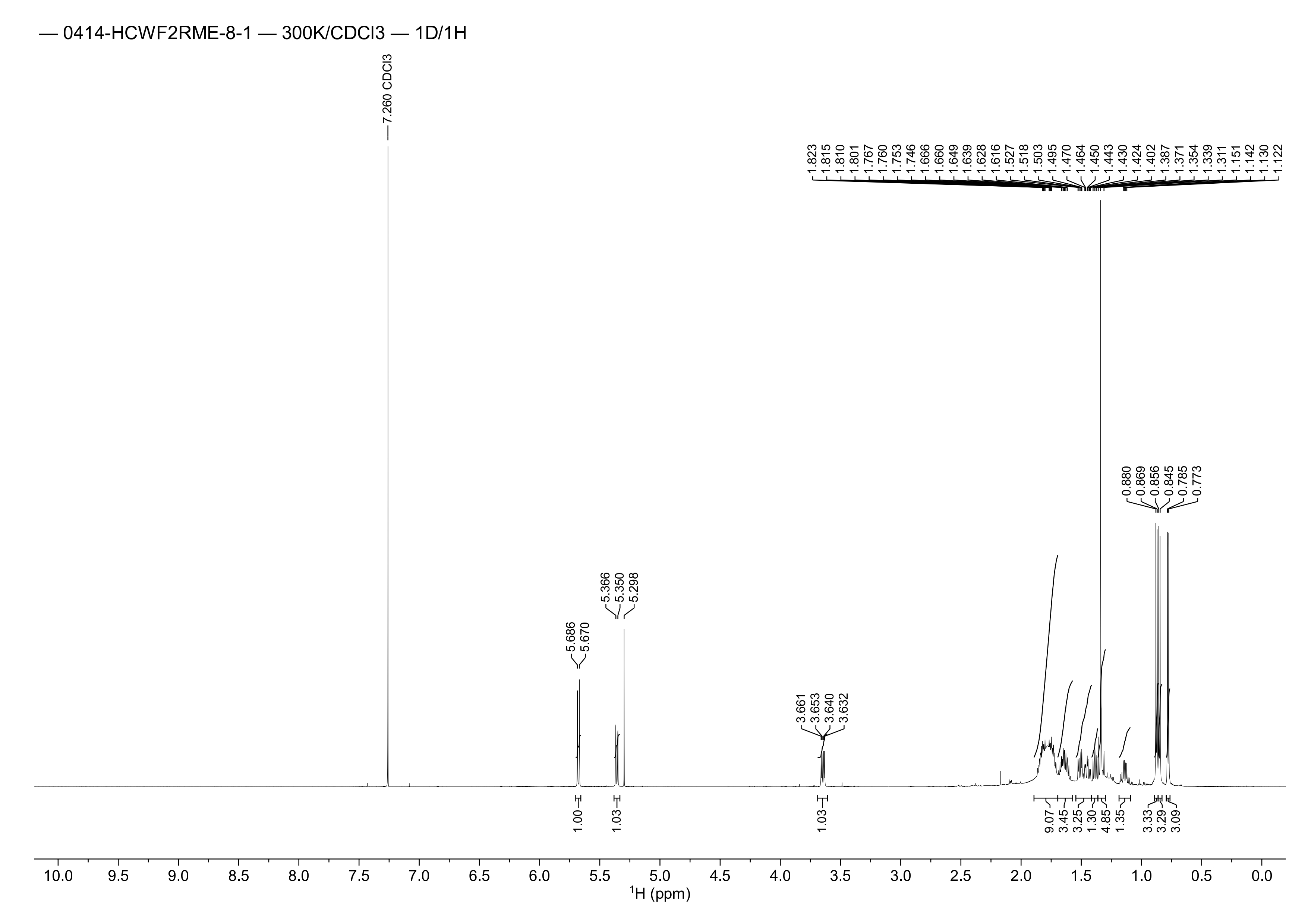


**Figure S3.** ^1^H NMR spectrum of compound **1** (CDCl_3_, 600 MHz)


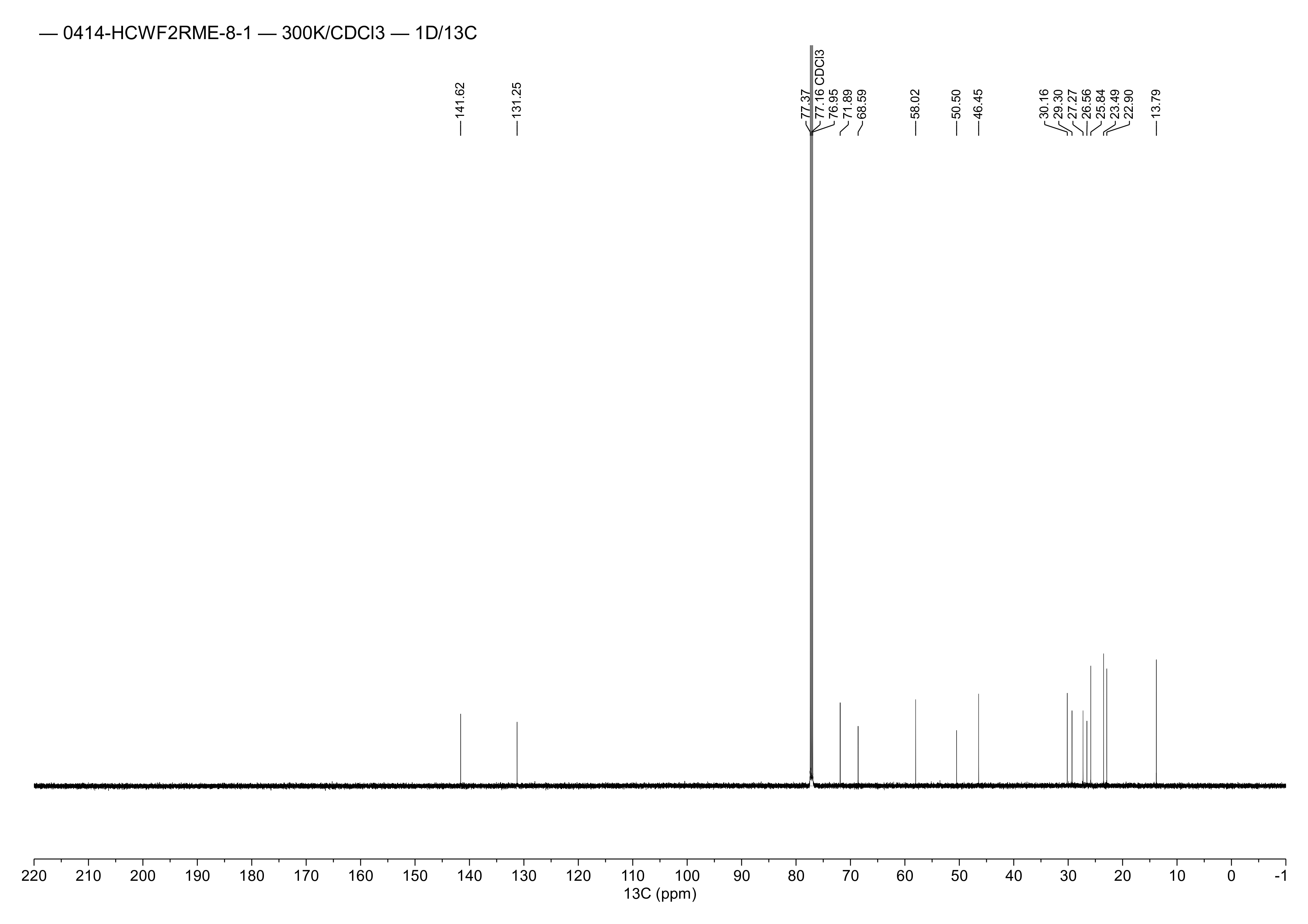


**Figure S4.** ^13^C NMR spectrum of compound **1** (CDCl_3_, 150 MHz)


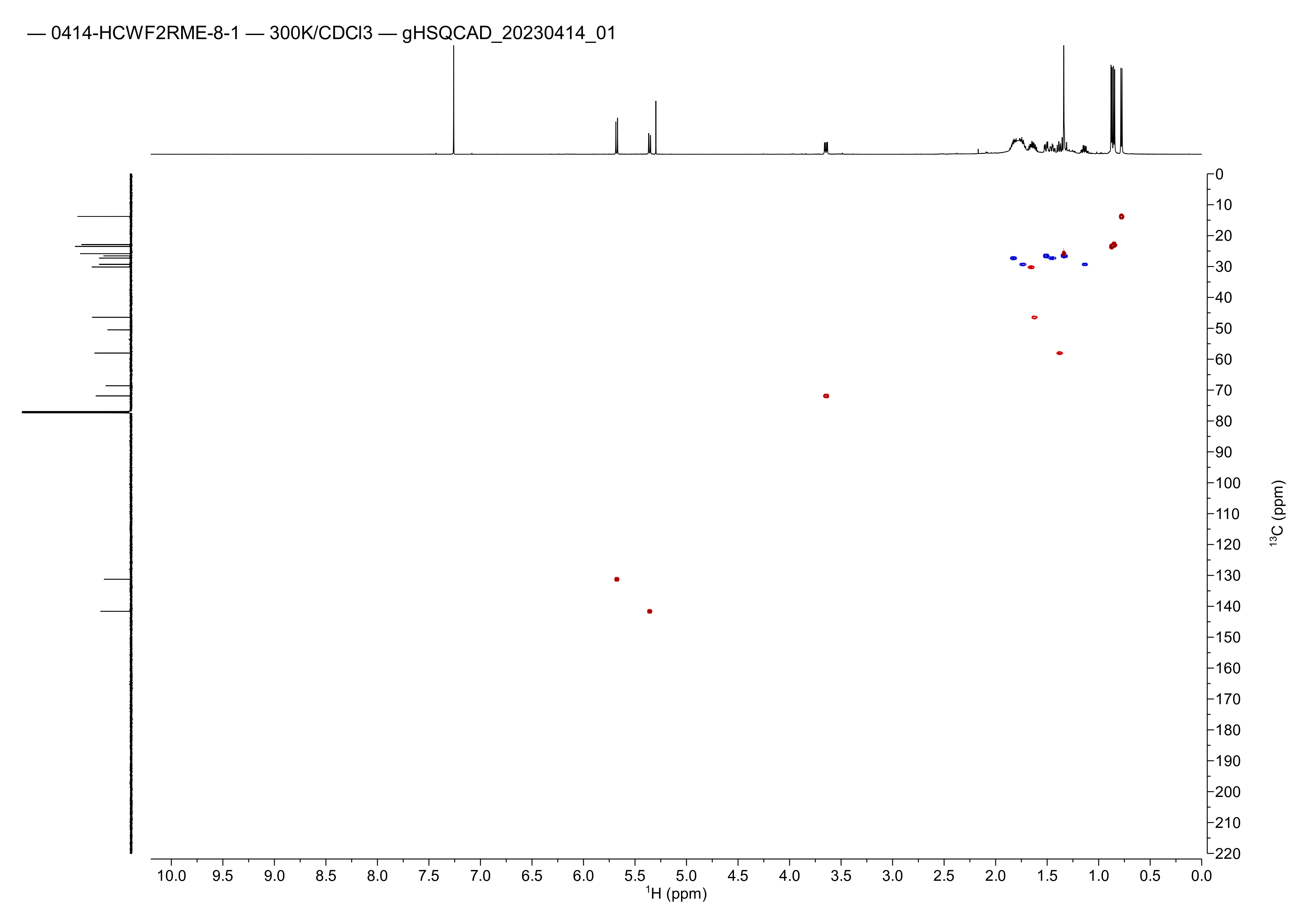


**Figure S5.** HSQC spectrum of compound **1**


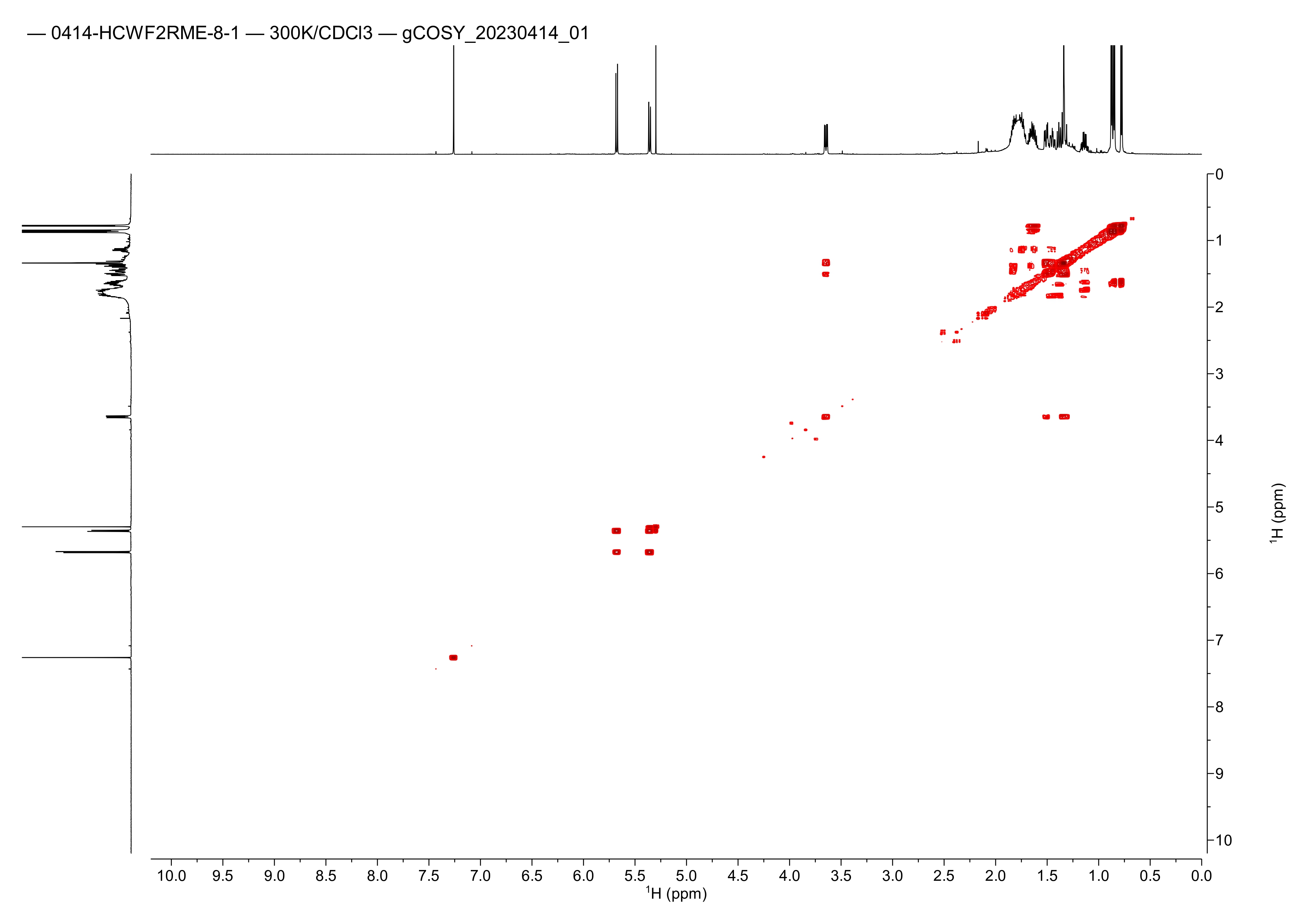


**Figure S6.** COSY spectrum of compound **1**


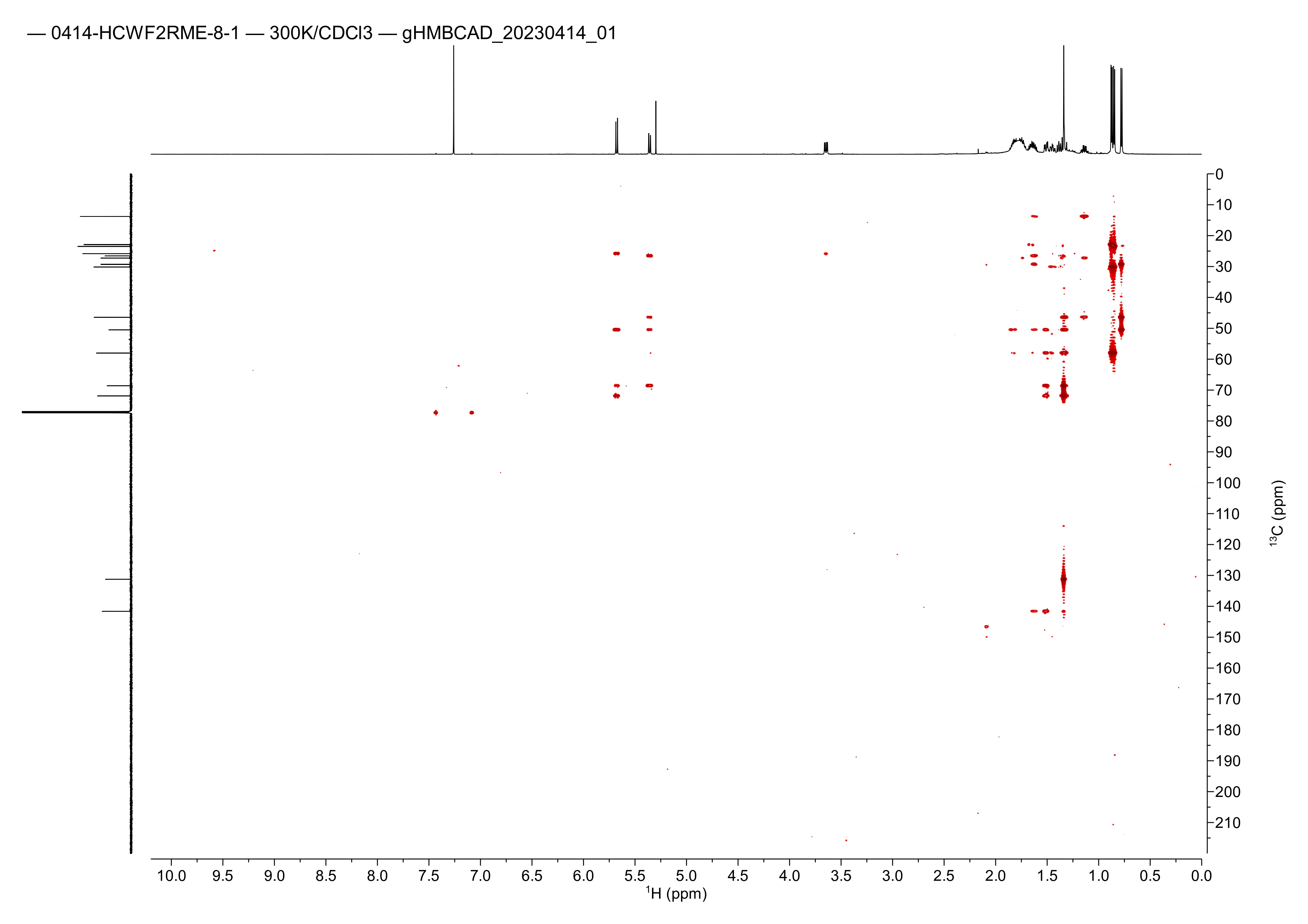


**Figure S7.** HMBC spectrum of compound **1**


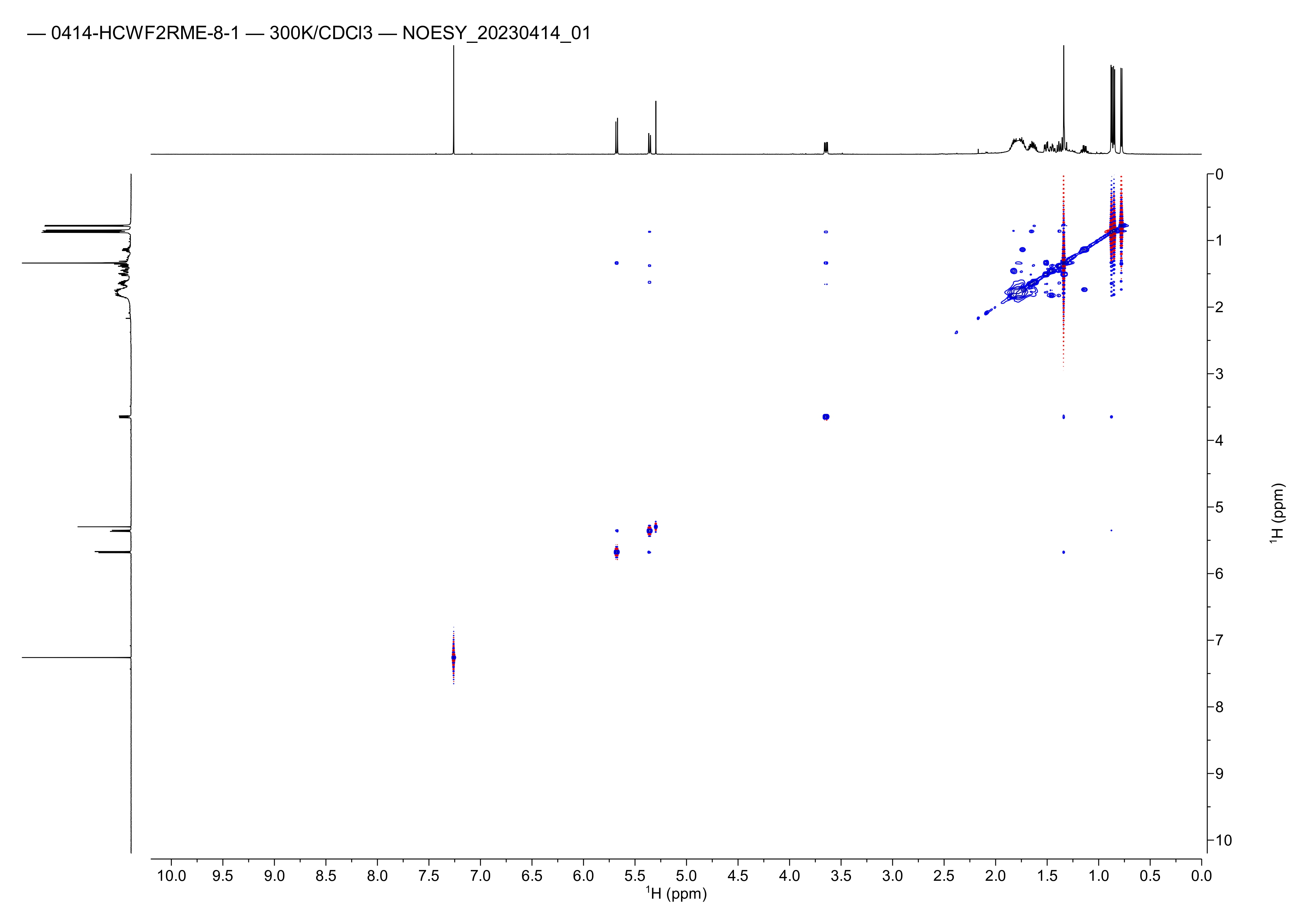


**Figure S8.** NOESY spectrum of compound **1**


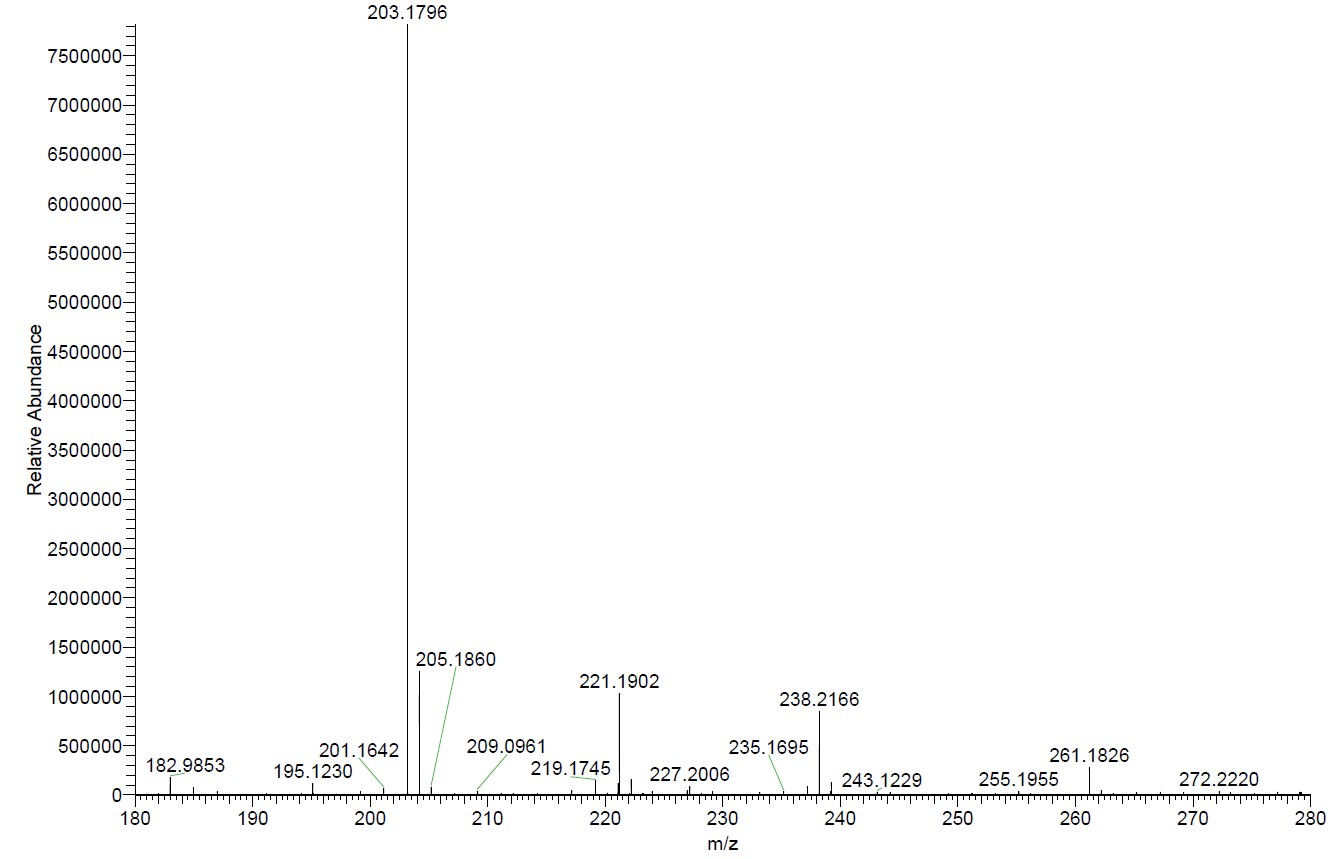


**Figure S9.** HRESIMS spectrum of compound **1**

**
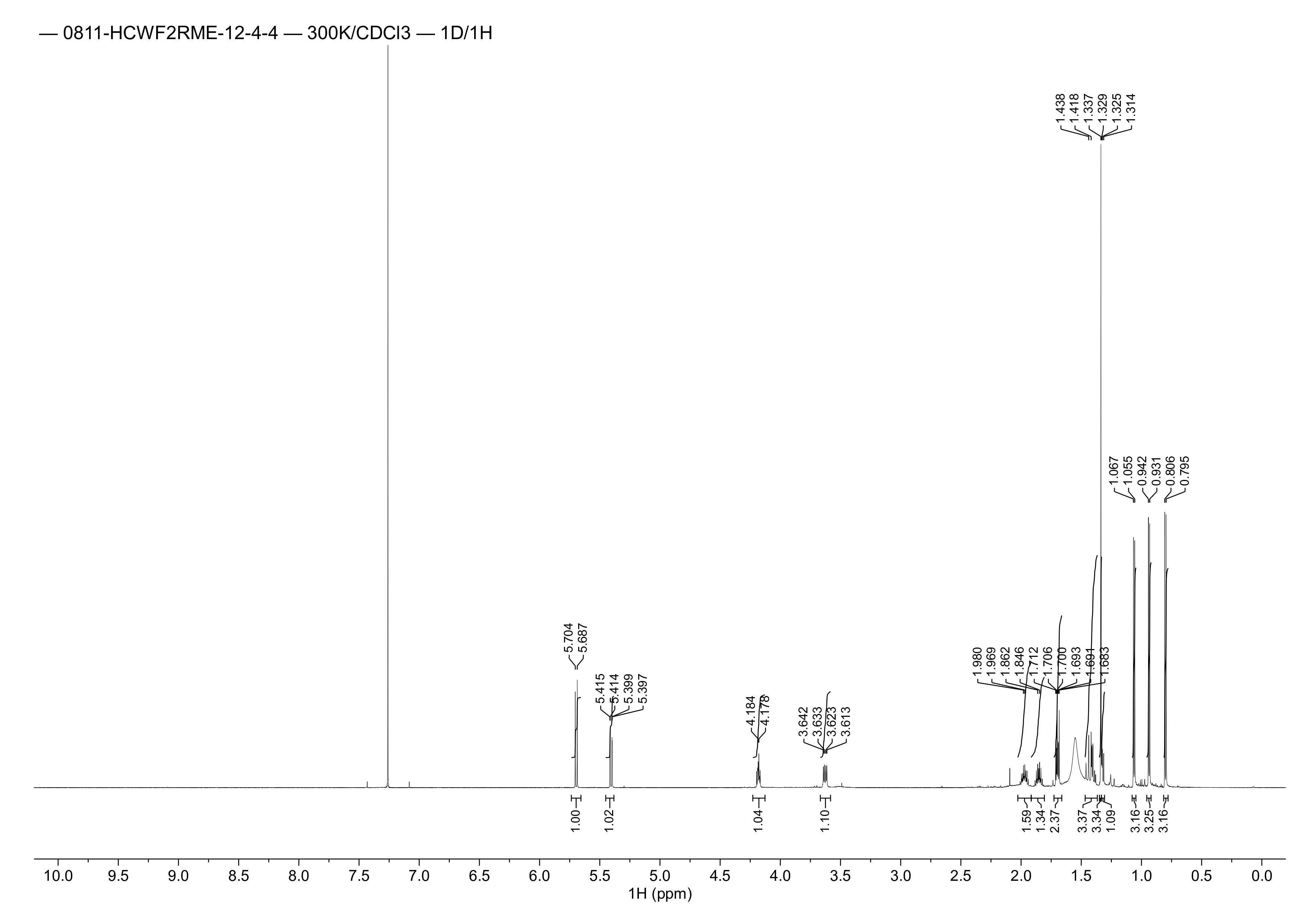
**

**Figure S10.** ^1^H NMR spectrum of compound **2** (CDCl_3_, 600 MHz)

**
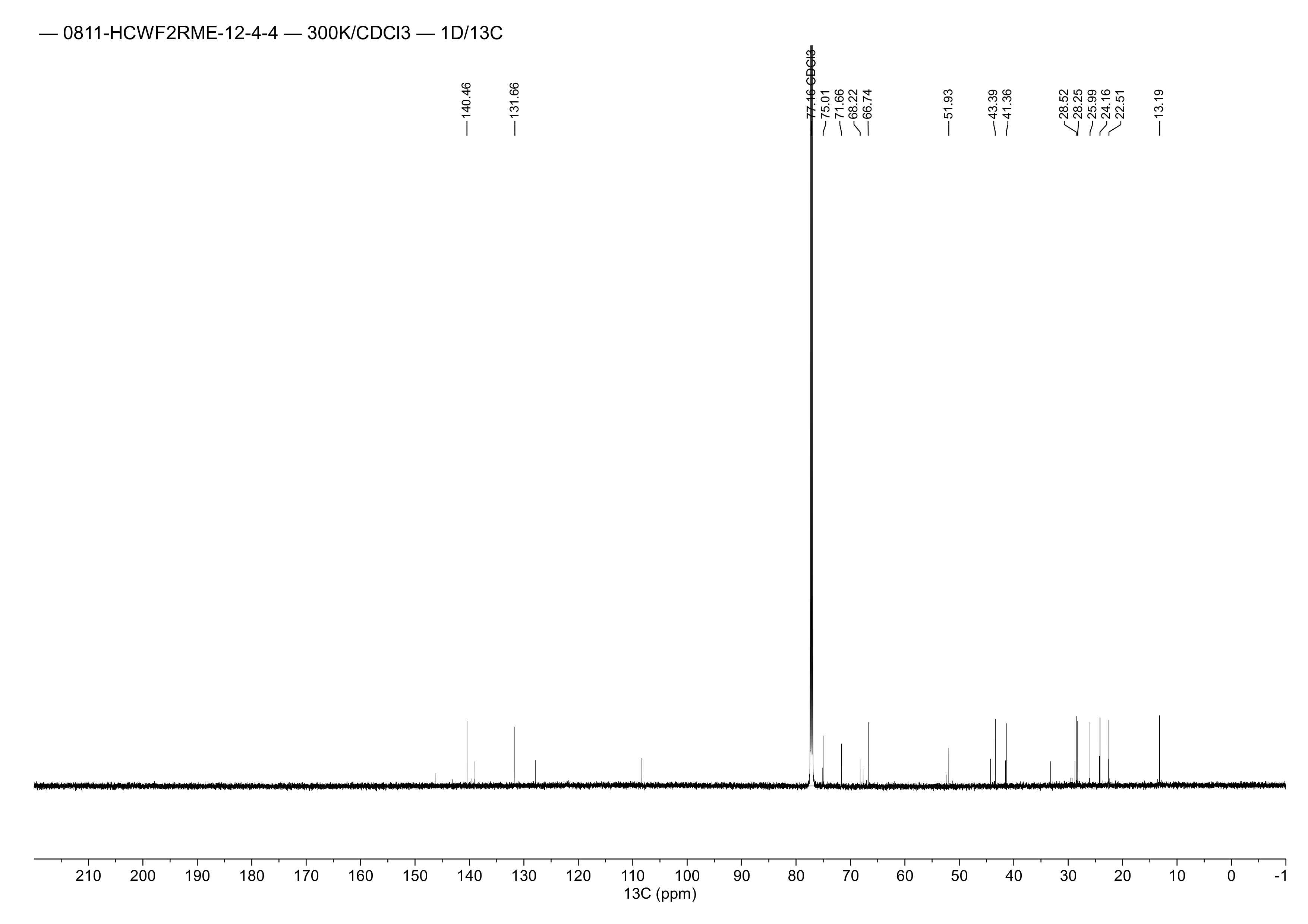
**

**Figure S11.** ^13^C NMR spectrum of compound **2** (CDCl_3_, 150 MHz)

**
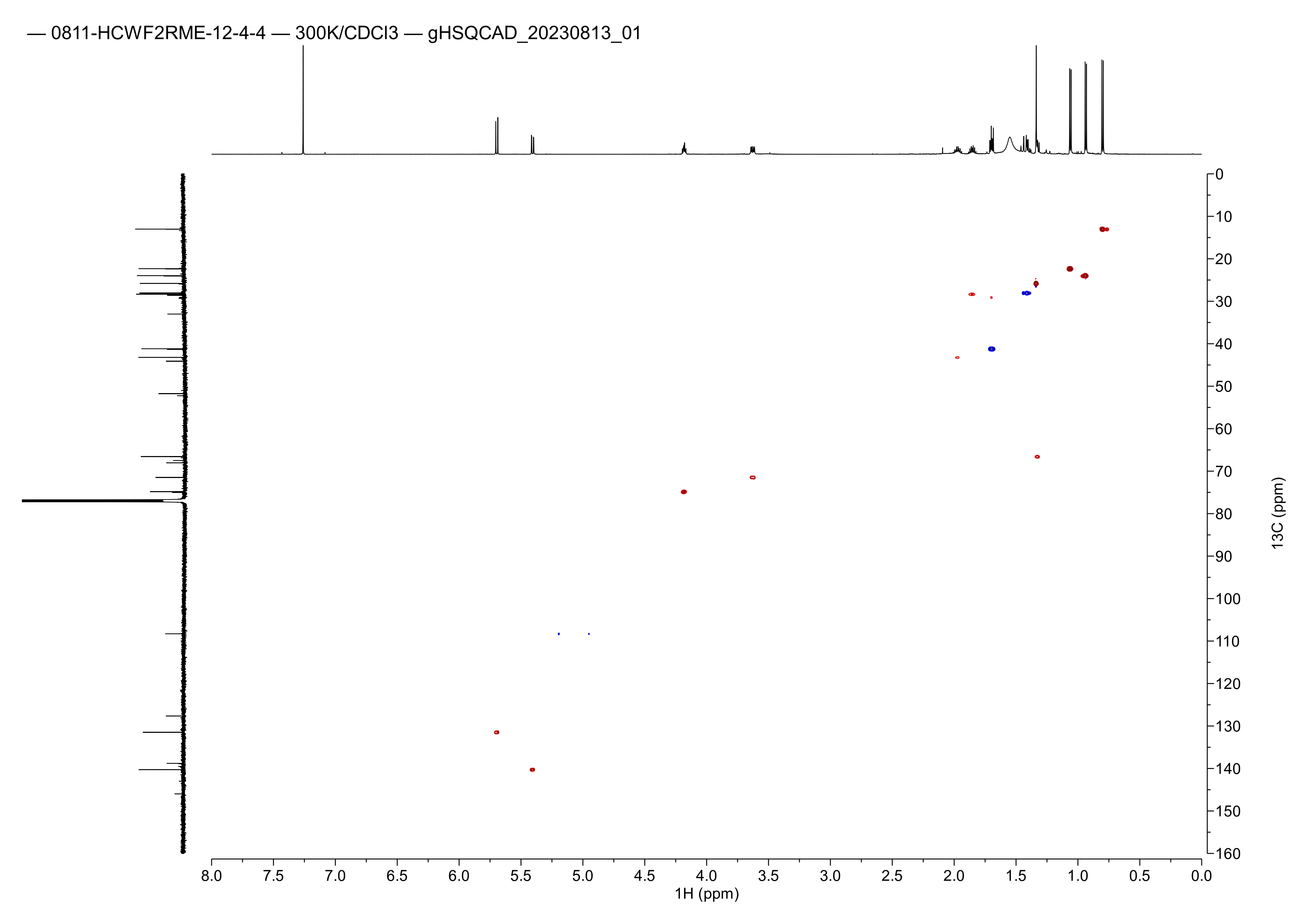
**

**Figure S12.** HSQC spectrum of compound **2**

**
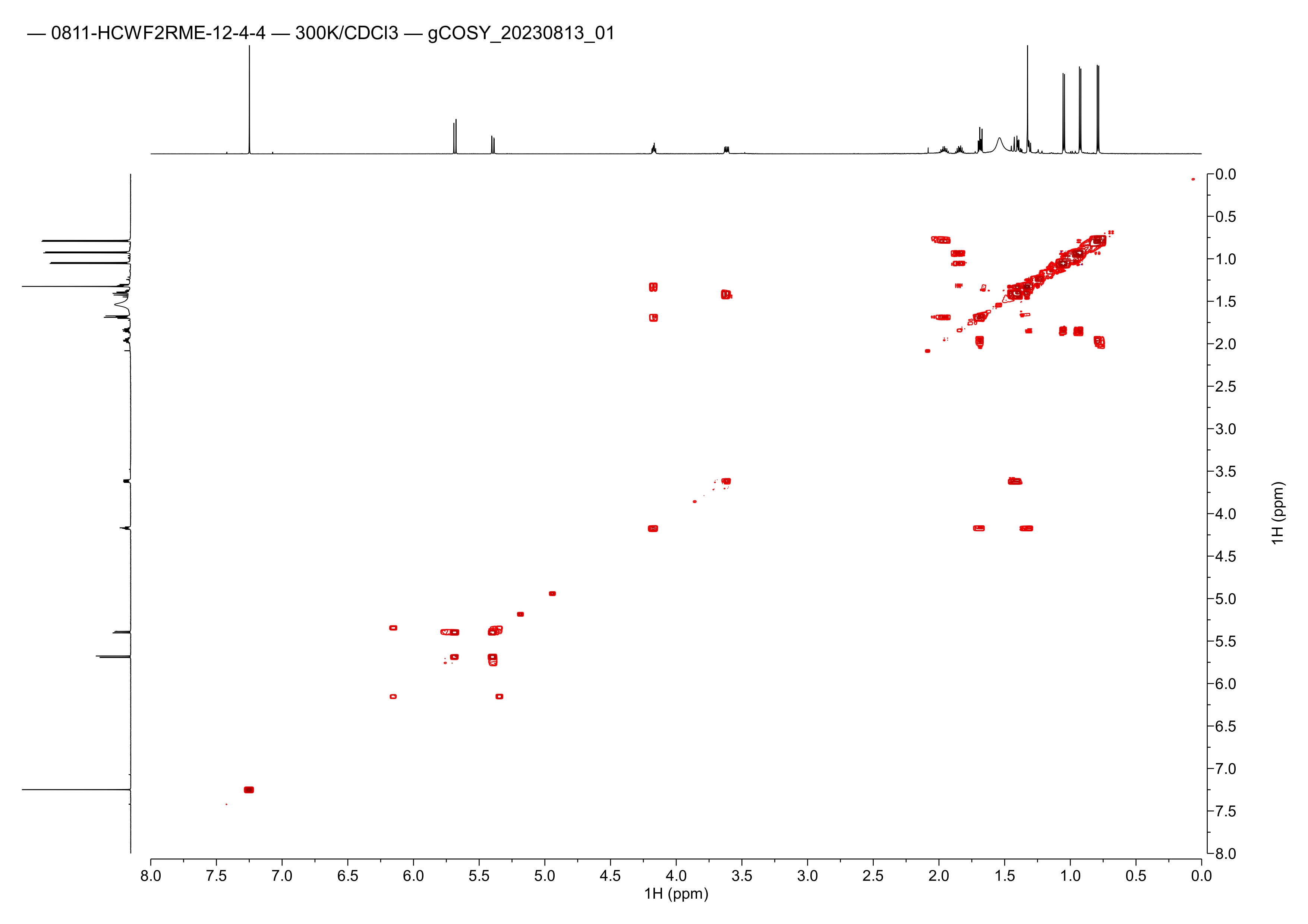
**

**Figure S13.** COSY spectrum of compound **2**

**
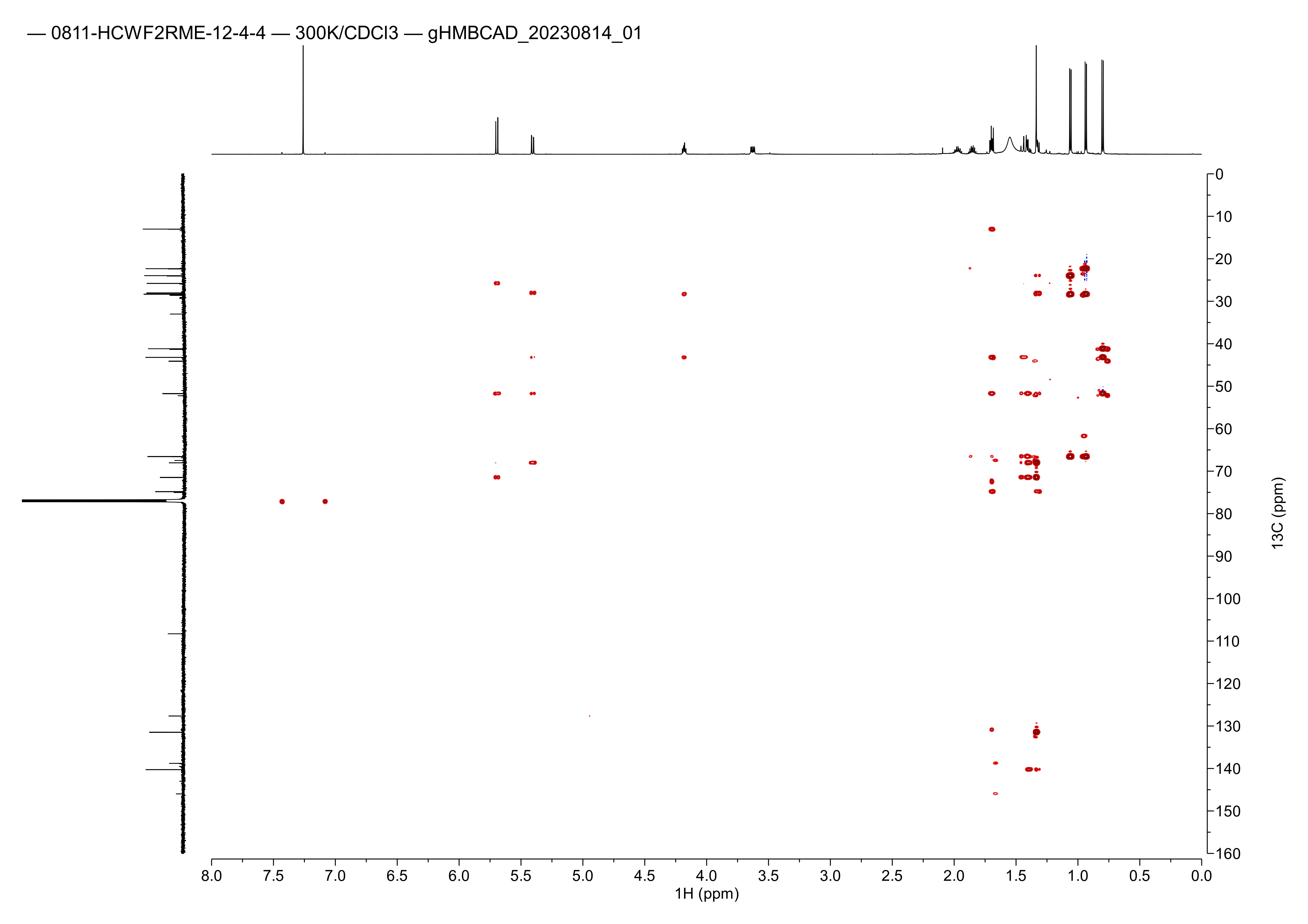
**

**Figure S14.** HMBC spectrum of compound **2**

**
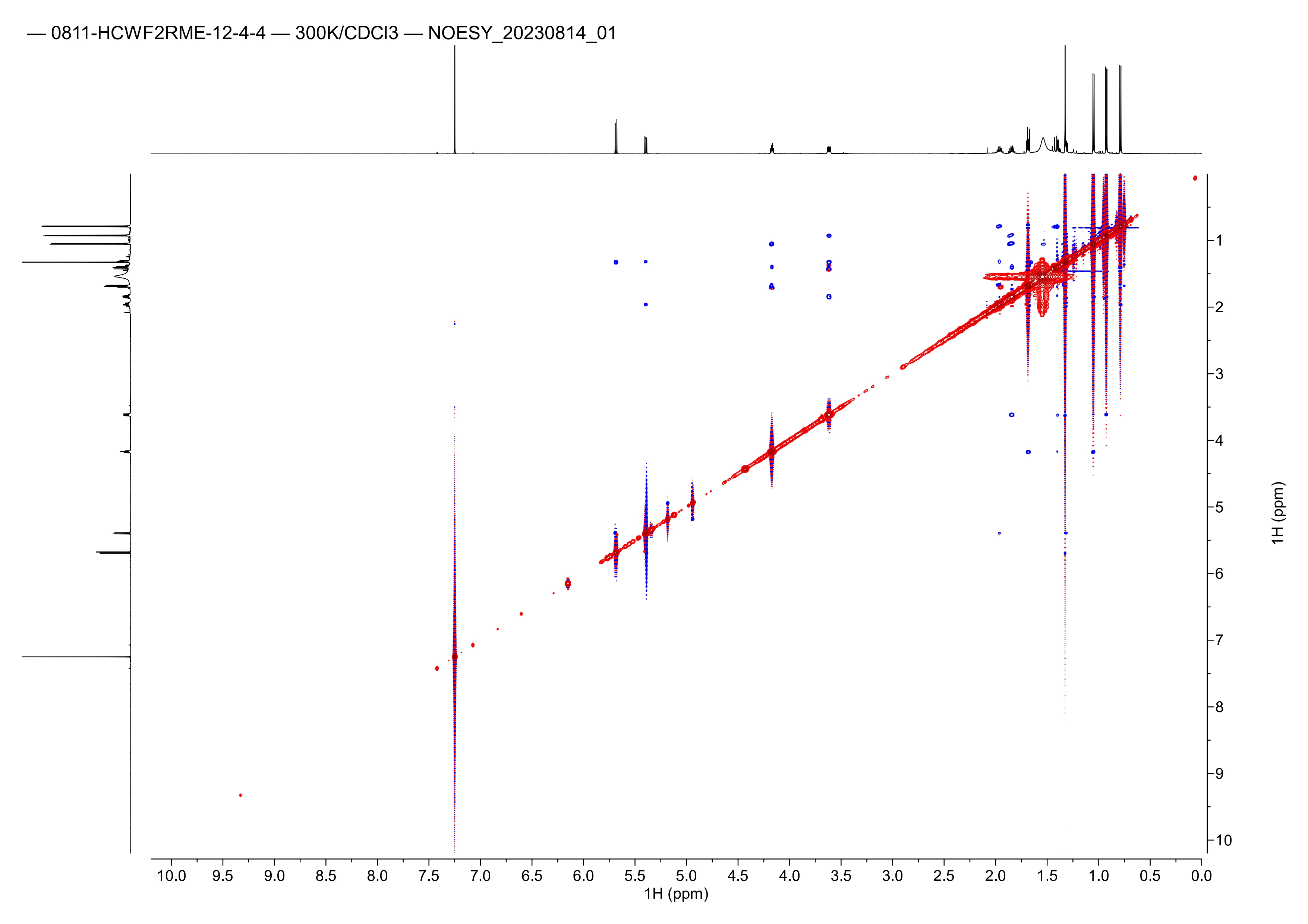
**

**Figure S15.** NOESY spectrum of compound **2**

**
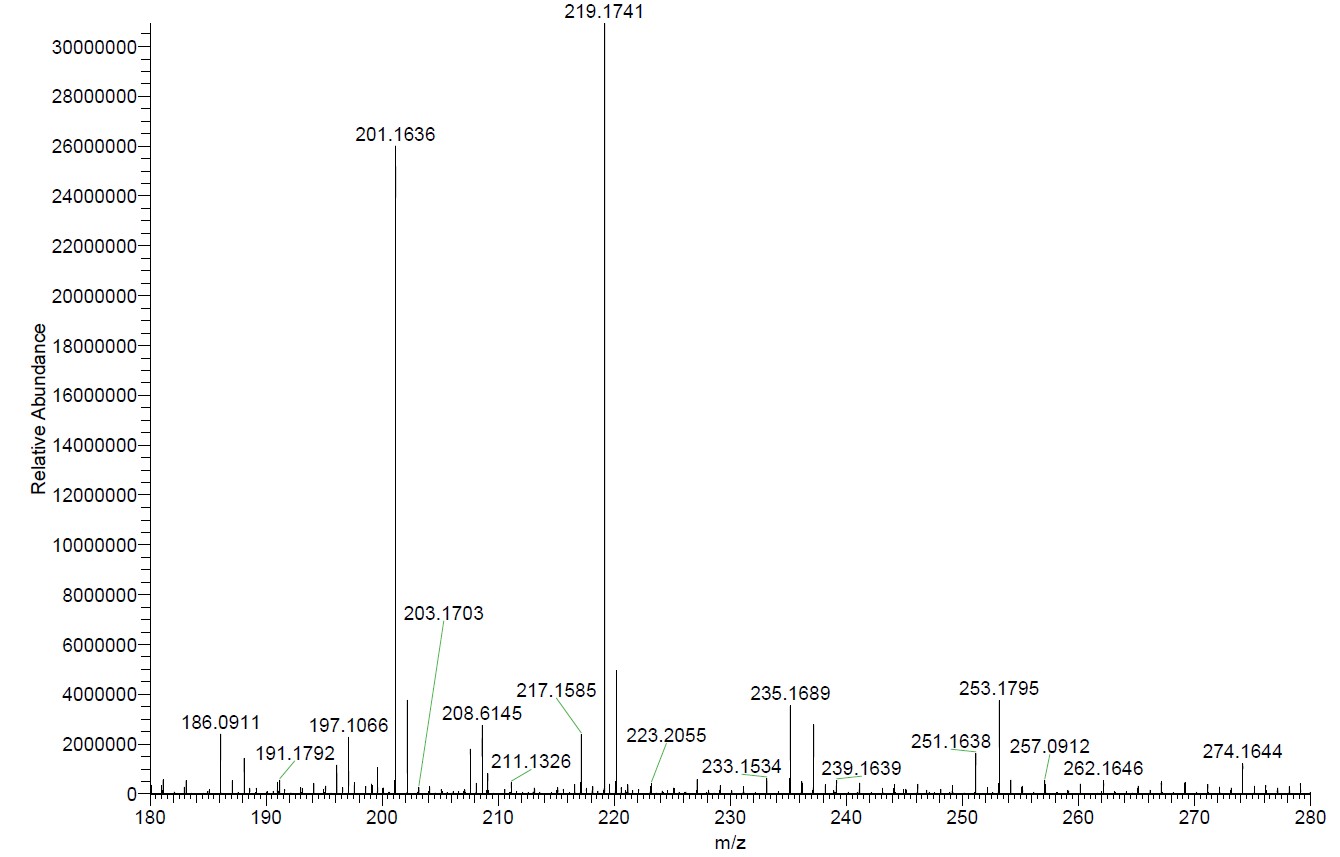
**

**Figure S16.** HRESIMS spectrum of compound **2**
